# Hepatocellular carcinoma‐infiltrating γδ T cells are functionally defected and allogenic Vδ2^+^ γδ T cell can be a promising complement

**DOI:** 10.1002/ctm2.800

**Published:** 2022-04-07

**Authors:** Wenjing He, Yi Hu, Dan Chen, Yijia Li, Dongmei Ye, Qiang Zhao, Li Lin, Xiaomin Shi, Ligong Lu, Zhinan Yin, Xiaoshun He, Yifang Gao, Yangzhe Wu

**Affiliations:** ^1^ Organ Transplantation Unit First Affiliated Hospital Sun Yat‐sen University Guangzhou Guangdong P.R. China; ^2^ Guangdong Provincial Key Laboratory of Organ Donation and Transplant Immunology The First Affiliated Hospital Sun Yat‐sen University Guangzhou Guangdong P.R. China; ^3^ Microbiology and Immunology Department School of Medicine Jinan University Guangzhou Guangdong P.R. China; ^4^ Zhuhai Institute of Translational Medicine Zhuhai People's Hospital (Zhuhai Hospital Affiliated with Jinan University) Jinan University Zhuhai Guangdong P.R. China; ^5^ The Biomedical Translational Research Institute Jinan University Guangzhou Guangdong P.R. China

**Keywords:** dysfunctional fingerprint, HCC, tumour Immunity, γδ T cell

## Abstract

In hepatocellular carcinoma (HCC), γδ T cells participate in mediating the anti‐tumour response and are linked with a positive prognosis. However, these cells can become pro‐tumoural in the tumour microenvironment (TME). We aimed to decipher the immune landscape and functional states of HCC‐infiltrating γδ T cells to provide fundamental evidence for the adoptive transfer of allogeneic Vδ2^+^ γδ T cells in HCC immunotherapy. We performed single‐cell RNA sequencing (scRNA‐seq) on γδ T cells derived from HCC tumours and healthy donor livers. Confocal microscopy, flow cytometry and a Luminex assay were applied to validate the scRNA‐seq findings. The γδ T cells in the HCC TME entered G2/M cell cycle arrest, and expressed cytotoxic molecules such as interferon‐gamma and granzyme B, but were functionally exhausted as indicated by upregulated gene and protein LAG3 expression. The γδ T cells in the HCC TME were dominated by the LAG3^+^Vδ1^+^ population, whereas the Vδ2^+^ γδ T population was greatly depleted. Moreover, glutamine metabolism of γδ T cells was markedly upregulated in the glutamine‐deficient TME. Both in vitro and in vivo experiments showed that glutamine deficiency upregulated LAG3 expression. Finally, our results indicated that ex vivo‐expanded Vδ2^+^ γδ T cells from healthy donor could complement the loss of T cell receptor clonality and effector functions of HCC‐derived γδ T cells. This work deciphered the dysfunctional signatures of HCC‐infiltrating γδ T cells in the HCC TME, providing scientific support for the use of allogeneic Vδ2^+^ γδ T cells in HCC cellular therapy.

## Graphical abstract

(1) HCC‐infiltrating γδ T cells lost T cell receptor (TCR) diversity and were G2/M cell cycle arrested.
(2) γδ T cell dysfunction in HCC was LAG3 dependent.
(3) Glutamine metabolism‐mediated γδ T cell dysfunction in HCC.
(4) Allogeneic Vδ2^+^ γδ T cells can complement functional deficiency of γδ T cells in HCC.

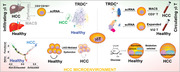

## 1 INTRODUCTION

Hepatocellular carcinoma (HCC) is the sixth leading cause of cancer‐related deaths worldwide^1^ and is infamously intransigent to chemotherapy.^2^ Currently, advances in HCC therapy, including an immune checkpoint molecule (e.g. PD1/PDL1) blockade strategy and adoptive transfer of immune cells [e.g. chimeric antigen receptor‐T cell (CAR‐T)] provide sturdy opportunities for HCC immunotherapy, which has been elegantly summarised by Sangro et al.^3^ Notably, Zhai and coworkers^4^ proposed glypican‐3 (GPC3)‐based CAR‐T for HCC therapy. Interestingly, GPC3 has also been engineered into γδ T cells to generate universal CAR‐T for HCC treatment.^5^ Although immune checkpoint blockade has achieved great progress in HCC therapy, the immune cell‐based strategy requires more intensive investigations. Since the HCC tumour microenvironment (TME) is highly heterogeneous, thoroughly deciphering of the immune landscape of the HCC TME is required for the development of more effective immune cell approaches [e.g. CAR‐T or T cell receptor (TCR)‐T] against HCC. Multi‐omics profiling^6^ and signatures of single immune cells^7^, ^8^, ^9^, ^10^, ^11^ of the HCC TME have greatly promoted understanding of the immune landscape of the complicated HCC TME, which will eventually facilitate the development of a novel immunotherapy protocol against HCC.

Previously, we have focused on the application of γδ T cells, specifically allogeneic Vδ2^+^ γδ T cells, to treat late‐stage cancer patients,^12^, ^13^ and demonstrated sound efficacy in HCC.^13^ However, to our knowledge, the fingerprints of γδ T cells in the HCC TME have not yet been analysed in detail. The liver is a rich source of γδ T cells.^14^ The γδ TCR is expressed by 6.8%–34% of liver CD3^+^ T cells.^15^ Intratumoural γδ T cells are considered a prognostic marker in pan‐cancer analysis.^16^ Importantly, the infiltration efficacy of γδ T cells might correlate with HCC progression and patient prognoses.^17^ Our clinical studies^12^, ^13^ showed that Vδ2 T cell therapy could significantly slow down the progression of liver cancer. Although γδ T cell infiltration was lower in HCC than in peri‐tumour tissues,^17^ the functional differences between γδ T cells in the HCC TME and normal liver tissue remain poorly understood. Thus, further characterising and distinguishing the heterogeneous immune fingerprints of infiltrating γδ T cells will reveal their functional complexity and transformation, and may lead to more effective HCC immunotherapies.

Here, we applied single‐cell RNA sequencing (scRNA‐seq) to examine the functional fingerprints of γδ T cells from liver perfusates of healthy donors and HCC patients. We explored the functional aspects of γδ T cells and attempted to establish correlations between gene expression patterns and corresponding protein expression phenotypes in the TME. Furthermore, we compared the genetic fingerprints of infiltrating γδ T cells from patients and healthy donors, demonstrating a loss of TCR diversity in HCC patients. Pseudo‐time analyses of HCC‐infiltrated γδ T cells showed exhausted and terminally differentiated features. The expression of stress response genes and cytotoxic genes was upregulated, indicating the complex nature of this population. Metabolic pathway analysis revealed a drastic alteration in γδ T cell metabolism in HCC compared to healthy livers, as evidenced by upregulation of glutamine metabolism, and significant downregulation of the oxidative phosphorylation (OXPHOS), glycolysis and fatty and amino acid metabolic pathways. Our data further suggest that Vδ2^+^ γδ T cells expanded from peripheral blood mononuclear cells (PBMCs) of healthy donors could complement the functional loss observed in HCC‐infiltrated γδ T cells.

## 2 MATERIALS AND METHODS

### 2.1 Ethics for liver sample collection

This study was conducted in accordance with the ethical principles stated in the Declaration of Helsinki. This study was fully approved by the First Affiliated Hospital of Sun Yat‐sen University ethical board [2018]073. All participants provided written informed consent before sample collection. Samples were collected either before or during liver transplantation at the First Affiliated Hospital of Sun Yat‐sen University (Guangzhou, China).

### 2.2 Cell enrichment from liver perfusate

To maintain the hepatic function of healthy donated liver, ex situ liver perfusion is a routine protocol before the liver transplantation for HCC patient, and the liver perfusate could be collected to enrich immune cells for hepatic immunological studies.^18^, ^19^ The standard routine perfusion procedures have been adopted by our Organ Transplantation Unit as well. In this project, the same methodology as reported^18^, ^19^ was used to enrich liver‐resident immune cells from the liver perfusate. The reason of using perfusate includes (1) the major purpose of this work was to compare functional difference between healthy liver tissue‐resident γδ T cells and HCC tumour‐resident γδ T cells, rather than tumour versus peri‐tumour, (2) the donated healthy liver cannot be sliced a piece off for investigation purpose, which would potentially impair the benefits of the patients. Therefore, we used perfusate rather than live tissue homogenate to enrich immune cells. In brief, the first 500 ml of the 1 L total perfusate was discarded and the second 500 ml was collected for mononuclear cell enrichment [Ficoll‐Paque PLUS (GE: 17‐1440‐03) density gradient]. For further in vitro validation, approximately 20 additional HCC patients and 20 healthy volunteers were recruited to collect peripheral blood (5–10 ml). This cohort study was approved by the ethical committee of the hospital. Ficoll‐Paque centrifugation isolation was also used for PBMC isolation from the peripheral blood of these additional samples.

### 2.3 CD3^+^CD161^+^ T cell enrichment using magnetic‐activating cell sorting

For scRNA‐seq, the cell number and viability were determined by a haemocytometer and Trypan Blue staining. Dead cells were removed using the Dead Cell Removal Kit (Miltenyi, 130‐090‐101) following the standard protocol. According to flow cytometry validation, the counts of γδ T cells in CD3^+^ T cells were too low to conduct 10×‐seq by γδ‐enrichment; thus, we first used CD3 and CD161 as markers for enrichment. Before magnetic‐activating cell sorting (MACS), we mixed three perfusate samples (each containing 1 × 10^7^ mononuclear cells) into a single pooled sample for the healthy liver and HCC perfusate, respectively. Subsequently, CD3^+^CD161^+^ T cells from the liver perfusates were harvested using the standard MACS protocol. The cells were immediately transferred into the 10× scRNA‐seq platform.

### 2.4 Selective expansion of Vγ9Vδ2 T cells from PBMCs

The isolated PBMCs from healthy donors were cultured in Roswell Park Memorial Institute (RPMI)‐1640 medium supplemented with 10% fetal bovine serum, zoledronate (50 μM, Sigma), recombinant human interleukin‐2 (100 IU/ml, Beijing Four Rings Bio‐Pharm Co.) and vitamin C (800 IU/ml, Sigma). Vγ9Vδ2 T cells with >90% purity were used in our experiments (e.g. in vitro functional phenotyping and scRNA‐seq).

### 2.5 scRNA‐seq

Four types of samples were used for scRNA‐seq, including CD3^+^CD161^+^ T cells enriched from the HCC liver perfusate (S#1) or healthy liver perfusate (S#2), γδ T cells enriched from the peripheral blood of HCC patients (S#3; blood samples were collected during the liver transplantation), and ex vivo‐expanded Vγ9Vδ2 T cells from healthy donors (S#4). Due to the restriction of tissue collection from the healthy donor liver, each sample (except for the ex vivo‐expanded Vγ9Vδ2 T cells) was thus pooled as a mixture that originated from three individuals. The scRNA‐seq was performed on the 10× Genomics platform, and the data were analysed with the Cell Ranger software pipeline (version 3.1.0). A total of 25 030 CD3^+^CD161^+^ T cells (8449 cells from HCC and 16 581 cells from the healthy liver) with a median of 824 genes and a mean 21 635 reads per cell were detected and analysed (S#1&2). Among these cells, 1622 cells were γδ T cells, with 173 cells from HCC (S#1) and 1449 cells from healthy liver (S#2), which were identified according to the TRDC^+^ phenotype. As for S#3, 13 938 cells with a median of 1326 genes and a mean of 38 349 reads per cell were measured (including 543 γδ T cells). Furthermore, to evaluate potential functional compensations of γδ T cells expanded from the PBMCs of healthy donors to HCC tumour‐infiltrated cells, we also performed scRNA‐seq on these ex vivo‐expanded γδ cells (S#4), which consisted of three separate samples: 13 523 cells with a median of 3796 genes and a mean of 41 548 reads per cell (10 383 γδ T cells); 14 586 cells with a median of 3794 genes and a mean of 39 782 reads per cell (11 201 γδ T cells); and 16 628 cells with a median of 3637 genes and a mean of 39 748 reads per cell (12 188 γδ T cells).

Differentially expressed genes (DEGs) were identified using the Seurat package. A *p*‐value <.05 and |log2foldchange| >1 or >0.58 was set as the threshold for significant differential expression. Gene Ontology (GO) and Kyoto Encyclopaedia of Genes and Genomes (KEGG) pathway enrichment analyses of DEGs were performed using R statistical software tools based on the hypergeometric distribution.

### 2.6 Effects of glutamine metabolism on inhibitory molecules and cytokines of γδ T cells

To partially mimic the TME in which glutamine is predominantly consumed by cancer cells, resulting in immune cells that are relatively glutamine deprived or insufficient, we established a glutamine deficiency condition in vitro to evaluate how glutamine affects the functional phenotypes of γδ T cells. Sample groups included: (1) γδ T cells cultured in routine RPMI‐1640 medium; (2) γδ T cells cultured in glutamine‐deficient RPMI‐1640 medium (Gln^−^); (3) γδ T cells treated with 1 mM d,l‐2‐aminoadipic acid (AAA), an inhibitor for intracellular glutamine synthesis; (4) Gln^−^ γδ T cells simultaneously treated with 1 mM AAA (Gln^−^ + AAA); and (5) γδ T cells treated with 1 mM CB839, an inhibitor of glutamine metabolism through GLS1. We further analysed the expression of inhibitory molecules (e.g. PD1, LAG3, TIM3) and cytokines [e.g. interferon‐gamma (IFN‐γ), tumour necrosis factor‐alpha (TNF‐α), granzyme B]. The treatment duration was 6 h unless otherwise indicated.

### 2.7 Transwell evaluation of the effect of HCC on γδ T cell function

Since glutamine is a favourable energy source for cancer cells, we conducted a transwell assay to mimic glutamine competition in the TME using an HCC cell line and γδ T cells. This in vitro experiment was designed to determine how the HCC growth environment affects the functional phenotypes of γδ T cells. The HCC cell line Bel7402 was used as the cancer cell model for this purpose. Bel7402 HCC cells were seeded in the apical chamber and γδ T cells were seeded in the basolateral chamber. The expression of inhibitory and cytotoxic molecules of γδ T cells were then analysed using flow cytometry after 24 h of incubation.

### 2.8 Statistics

Statistical analyses and graphing were performed using GraphPad Prism software. Experimental replicates and sample sizes are given in the corresponding figure legends. All results are expressed as mean ± standard error of the mean. Statistical significance was calculated using the paired or unpaired Student's *t*‐test methodology. *p*‐Values of less than or equal to .05 were identified as significant in all analyses.

## 3 RESULTS

### 3.1 Sample and patient characteristics

We used liver perfusates for scRNA‐seq, which raises the concern of whether they truly represent the liver microenvironment. To address this issue, we analysed the γδ T cell percentage in overall T cells among PBMCs, perfusates, and liver tissues collected from 12 patients. We found similar γδ T cell percentages between tissues and perfusates from the same patient (Supporting Information S1A). Immune cells extracted from liver perfusates, rather than regional tissues, can better represent the immune microenvironment of whole livers. Thus, we performed our scRNA‐seq analyses on liver perfusates. Furthermore, due to the limited counts of γδ T cells in perfusates, we pooled three samples each to obtain a single sample for both HCC and healthy liver tissues. All three patients had late‐stage HCC (stages III and IV) and their perfusates had similar γδ T cell proportions. Moreover, all three patients had hepatitis B virus (HBV)‐related HCC (Supporting Information S1B).

### 3.2 Infiltrating γδ T cell clustering and subtype analyses

**FIGURE 1:**
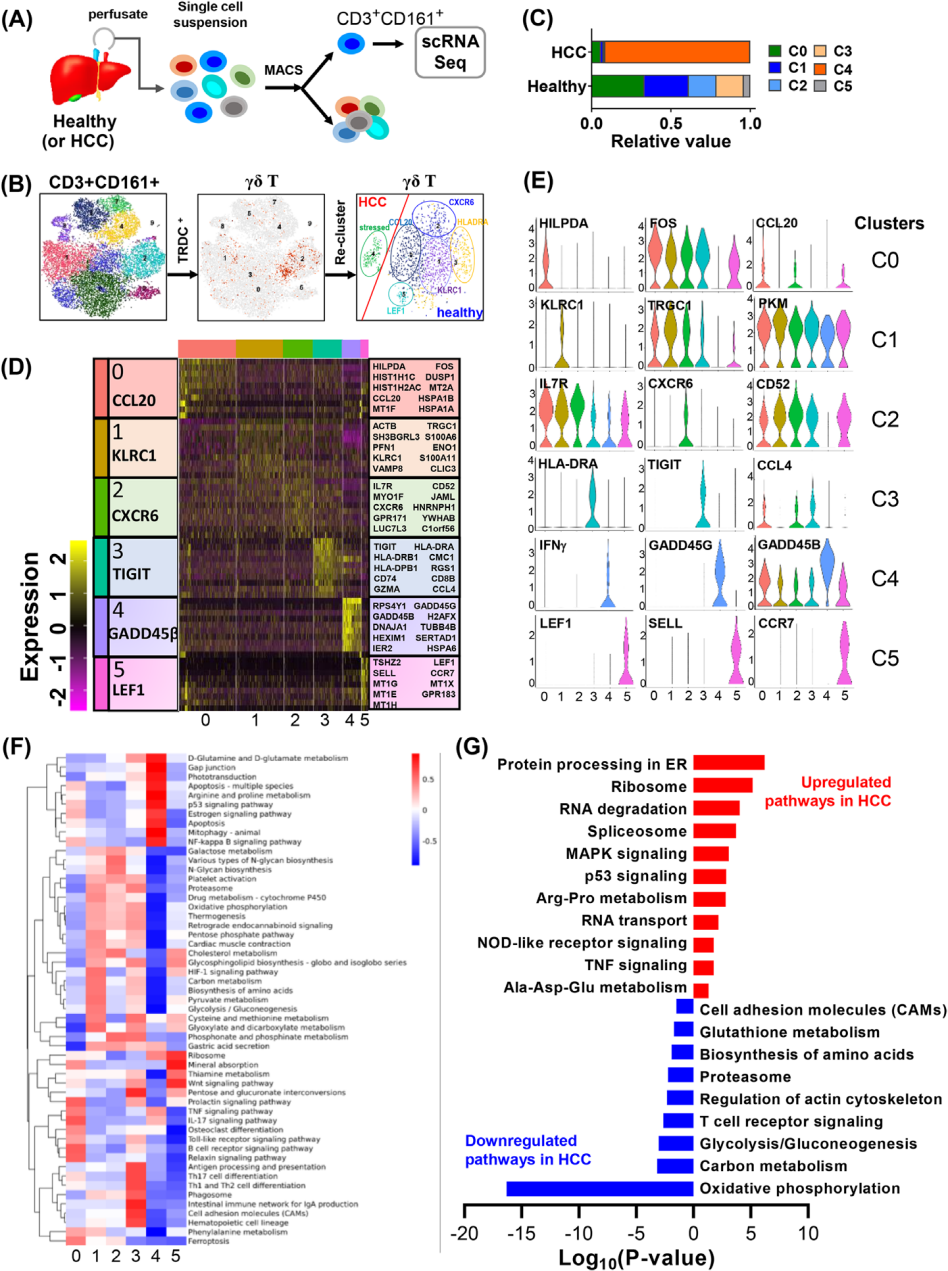
Profiling of tumour‐infiltrating γδ T cells in hepatocellular carcinoma and healthy livers with single‐cell RNA sequencing. (A) Schematic overview of the experiment workflow. (B) Two‐dimensional t‐distributed stochastic neighbourhood embedding (t‐SNE) plot visualisation of CD3^+^CD161^+^ T cell clusters from perfusate of hepatocellular carcinoma (HCC) livers and healthy donor livers (*n* = 3, respectively). Further extraction of the γδ T cell population was achieved using the TRDC^+^ marker, followed by the 2D t‐SNE projection of the re‐clustered γδ T cell population. (C) Relative composition of clusters of infiltrating γδ T cells in HCC and healthy liver perfusates. (D) Heatmap from single‐cell analysis with cells grouped into clusters according to top 10 marker genes. (E) Violin plots showing the expression profile of top marker genes from each cluster. (F) Gene set enrichment analyses showing the most differentially regulated functional pathways of each γδ T cell cluster. (G) Representative up‐ and downregulated pathways in HCC‐infiltrating compared with in donor liver‐infiltrating γδ T cells (*p* < .05)

To identify HCC‐infiltrating γδ T cell functional fingerprints, we utilised gel bead‐based scRNA‐seq (10× Genomics, Figure 1A). scRNA‐seq analyses were performed for peripheral and tissue‐infiltrating γδ T cells from HCC patients and healthy donors (Figure 1A,B). The initial clustering selected cells co‐expressing the surface markers CD3 and CD161, representing conventional CD4^+^, CD8^+^ and innate‐like T cells, including γδ T, invariant natural killer T and mucosal‐associated invariant T cells. We applied the TRDC marker, which is exclusively expressed in γδ T cells, and identified six unique γδ T cell clusters based on their gene expression profiles (Figure 1B,D,E). Clusters 0, 1, 2, 3 and 5 predominantly comprised liver‐infiltrating γδ T cells from healthy donor livers (S#2), whereas cluster 4 almost exclusively comprised infiltrating γδ T cells from HCC tumour (S#1) (Figure 1C). The top 10 DEGs in each cluster are shown in Figure 1D, and the major functional enrichment of individual clusters is provided in Figure S1.

The γδ T cells from the first cluster (C0‐*CCL20*, ∼33.33%) were the dominant population in the healthy liver and exclusively expressed effector memory T cell genes such as *CCL20*, *CCL3*, *IL7R* and *CD69*. The second cluster (C1‐*KLRC1*, ∼27.95%) was also highly populated in the healthy livers and was characterised by the effector T cell marker *KLRC1*. The third cluster (C2‐*CXCR6*, ∼17.39%) highly expressed *FYN*, *IL7R*, *CXCR6* and *CD52*, thus likely representing a memory T cell subset. The fourth cluster (C3‐*TIGIT*, ∼17.18%) was distinguished by high expression of the exhaustion marker *TIGIT* and functional markers *GZMK*, *GZMA*, *GZMH*, *GZMM*, *KLRC2* and *NKG7*. The fifth cluster (C4‐*GADD45β*) was almost solely detected in tumour samples (∼91.33% of total HCC‐infiltrating γδ T cells) and highly expressed stress marker genes such as *GADD45γ* and *GADD45β*, along with the exhaustion marker gene *LAG3* and cytotoxicity genes such as *NKG7*, *GNLY*, *GZMB* and *IFNG*, representing an exhausted, yet cytotoxic, population within HCC tumours. The last cluster (C5‐*LEF1*, ∼4.07%) was only detected in healthy livers and strongly expressed naïve T cell markers. Violin plots of selected top marker gene expression patterns are shown in Figure 1E. These results provide direct evidence for the distribution of γδ T cell populations in healthy livers and a loss of diversity in HCC patients. The most significantly altered KEGG pathways in C4 included drastic upregulation of the glutamine metabolism and apoptosis pathways, and downregulation of TCR signalling and central metabolic pathways such as glycolysis and OXPHOS, implying metabolic reprogramming and the loss of effector cell function of intratumoural γδ T cells (Figure 1F,G).

### 3.3 Loss of TCR diversity in HCC‐infiltrating γδ T cells

**FIGURE 2:**
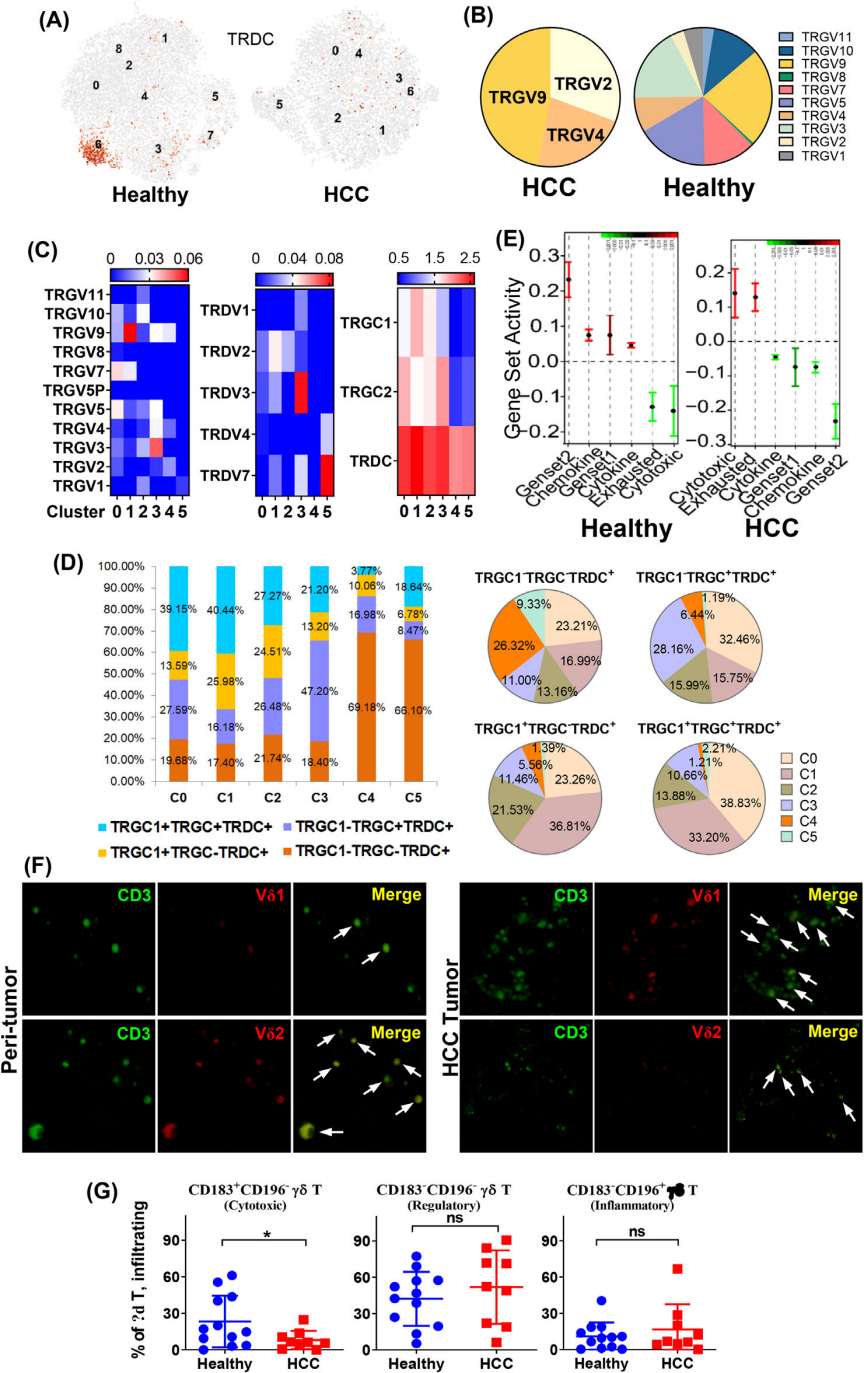
T cell receptor distribution and clonality analysis. (A) TRDC^+^ γδ T cells highly localised to the cluster 6 of CD3^+^CD161^+^ T cells in healthy livers, whereas TRDC^+^ γδ T cells showed a disperse distribution in clusters of CD3^+^CD161^+^ T cells in hepatocellular carcinoma (HCC+) tumours. (B) Loss of T cell receptor gamma variable (TRGV) diversity in HCC+ tumours compared with in healthy livers. (C) TRGV, T cell receptor gamma constant (TRGC), and T cell receptor delta variable (TRDV) gene expression patterns in clusters 0–5. (D) T cell receptor clone type and size comparisons among clusters. (E) Various gene set activity analyses of γδ T cells in healthy and HCC+ livers. Gene set 1 and 2 correspond to Vδ1 and Vδ2 gene sets, respectively. (F) Confocal visualisation of infiltrating Vδ1^+^ and Vδ2^+^ γδ T cells in both HCC tumour tissue and peri‐tumour tissue (60X oil immersion lens [OIL]). (G) Percentage and functional subtyping on γδ T cells in healthy and HCC+ livers using CD183 (CXCR3) and CD196 (CCR6). CD183^+^CD196^−^, CD183^−^CD196^−^ and CD183^−^CD196^+^ γδ T cells are considered as the cytotoxic (Th1), regulatory (Th2) and inflammatory (Th17) populations, respectively (healthy livers, *n* = 12; HCC livers, *n* = 9). ns, no significance; *p < .05

TCR diversity is inversely correlated with tumour progression.^20^ We also observed significant loss of TRDC^+^ γδ T cells in HCC livers (Figure 2A). Further analysis showed a drastic loss in T cell receptor gamma variable (TRGV) diversity in HCC tumour‐infiltrating γδ T cells compared with that in healthy controls (Figure 2B). Assessment of TRGV, T cell receptor gamma constant (TRGC) and T cell receptor delta variable (TRDV) variant expression patterns in γδ T cell clusters demonstrated a universal loss of TCR diversity in C4 (Figure 2C). TCR clono‐typing analyses demonstrated that >69% of γδ T cells in C4 expressed only one detectable TCR gene (Figure 2D).

We compared the published gene signatures of Vδ1 and Vδ2 populations (denoted as gene set 1 and 2, respectively)^21^ and observed that HCC‐infiltrating γδ T cells more closely resembled the Vδ1 expression profile (Figure 2E). Nonetheless, the overall enrichment of both Vδ1 and Vδ2 gene set activities was lower than that in the healthy controls, implying an overall loss of TCR diversity. We applied confocal microscopy to visualise the infiltrating Vδ1^+^ and Vδ2^+^ γδ T cells in peri‐tumour and tumour tissues (Figure 2F), which showed similar frequencies of Vδ1^+^ and Vδ2^+^ γδ T cells in the peri‐tumour tissue, and lower counts of infiltrating Vδ2^+^ γδ T cells in the tumour tissue (Figure S2A). Flow cytometry of γδ T cell subtypes based on Th1, Th2 and Th17 chemokine expression [CD183 (CXCR3) and CD196 (CCR6)]^22^ in liver perfusates of HCC patients and healthy donors indicated a significant decrease in the Th1 population in HCC‐infiltrating γδ T cells, implying the loss of cytotoxic functions (Figure 2G). Previous studies indicated that Vδ2^+^ behaved as a cytotoxic (Th1‐like) subtype; however, the role of Vδ1^+^ in tumour development is controversial. Our results suggested that the cytotoxic nature of γδ T cells was compromised in HCC livers.

Similar to HCC tumour‐infiltrating γδ T cells, the percentage of circulating Vδ2^+^ γδ T cells (cytotoxic) in the peripheral blood of liver cancer patients was significantly reduced, whereas that of circulating Vδ1^+^ γδ T cells (regulatory) strikingly increased. This led to a significant reduction in the Vδ2/Vδ1 ratio in liver cancer patients (Figure S2B). Based on the expression levels of CD183 and CD196, we found a statistically significant decrease in both cytotoxic and regulatory functions in HCC patients (Figure S2C). The loss of TCR diversity might lead to the loss of functional diversity in γδ T cells and their responsiveness in HCC patients, both in the TME and circulatory blood.

### 3.4 Developmental trajectory and cluster differentiation of γδ T cells

**FIGURE 3:**
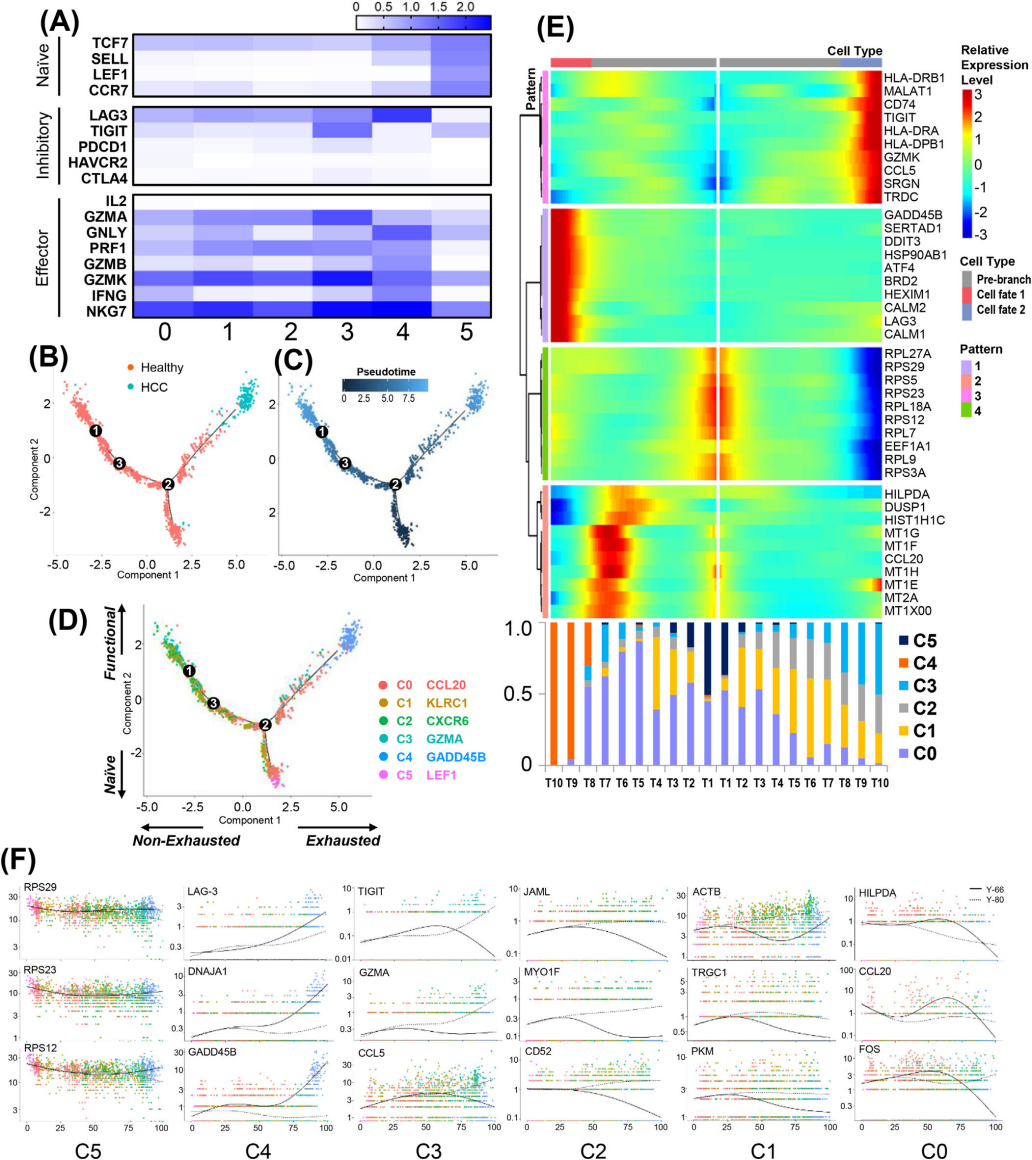
Mapping developmental trajectory by pseudo‐time state transition and RNA velocity analysis. (A) Heat map showing the gene expression of naïve, effector and inhibitor‐related T cell markers in each cluster. (B) Monocle prediction of cluster developmental trajectory with pseudo‐time. (C) Pseudo‐time profile of infiltrating γδ T cells from all clusters from hepatocellular carcinoma (HCC) and healthy controls. (D) RNA velocity profile of infiltrating γδ T cells from all clusters from HCC and healthy controls. (E) Differentially expressed genes (rows) along pseudo‐time were clustered hierarchically and generate four unique gene modules. The top 10 genes in each expression profile are shown. The corresponding cell population distribution profile is also overlaid with the gene expression profile to correlate cluster distribution and the respective transcriptomic profile. (F) The pseudo‐time trajectory of each representative functional gene in each cluster

To analyse the developmental trajectory of γδ T cells, we defined naïveness, cytotoxicity, exhaustion and non‐exhaustion scales in the two‐dimensional pseudo‐time plot based on previously defined gene signatures (Figure 3A,B).^23^, ^24^ Monocle showed a branched structure (Figure 3C), with C4 being the most exhausted cluster and C1–C3 representing effector clusters. The C0 cluster spread across all branches, suggesting a transitional intermediate functional state. The C5 cluster was defined as naïve T cells. RNA velocity^25^ analyses identified a strong directional flow from C5 towards either highly functional C1–C3 or transitional C0 and no reversed progression from C4 to other clusters (Figure 3D).

By imposing the cluster distribution in each cell subgroup, we identified the main expression patterns both in clusters and hypothetical cluster transitions. This produced four unique expression patterns (Figure 3E,F). C4, mainly originating from HCC samples, exhibited a pattern‐1 expression profile, mainly comprising T10 and T9 cell subgroups, whereas C5, the origin for all clusters, dominated the T1 subgroups. Clear cluster transitions were observed from C5 to C0 and from C0 to C1–C3, validating our observations shown in Figure 3D.

### 3.5 HCC‐infiltrating γδ T cells show reduced proliferation and G2/M cell cycle arrest

**FIGURE 4:**
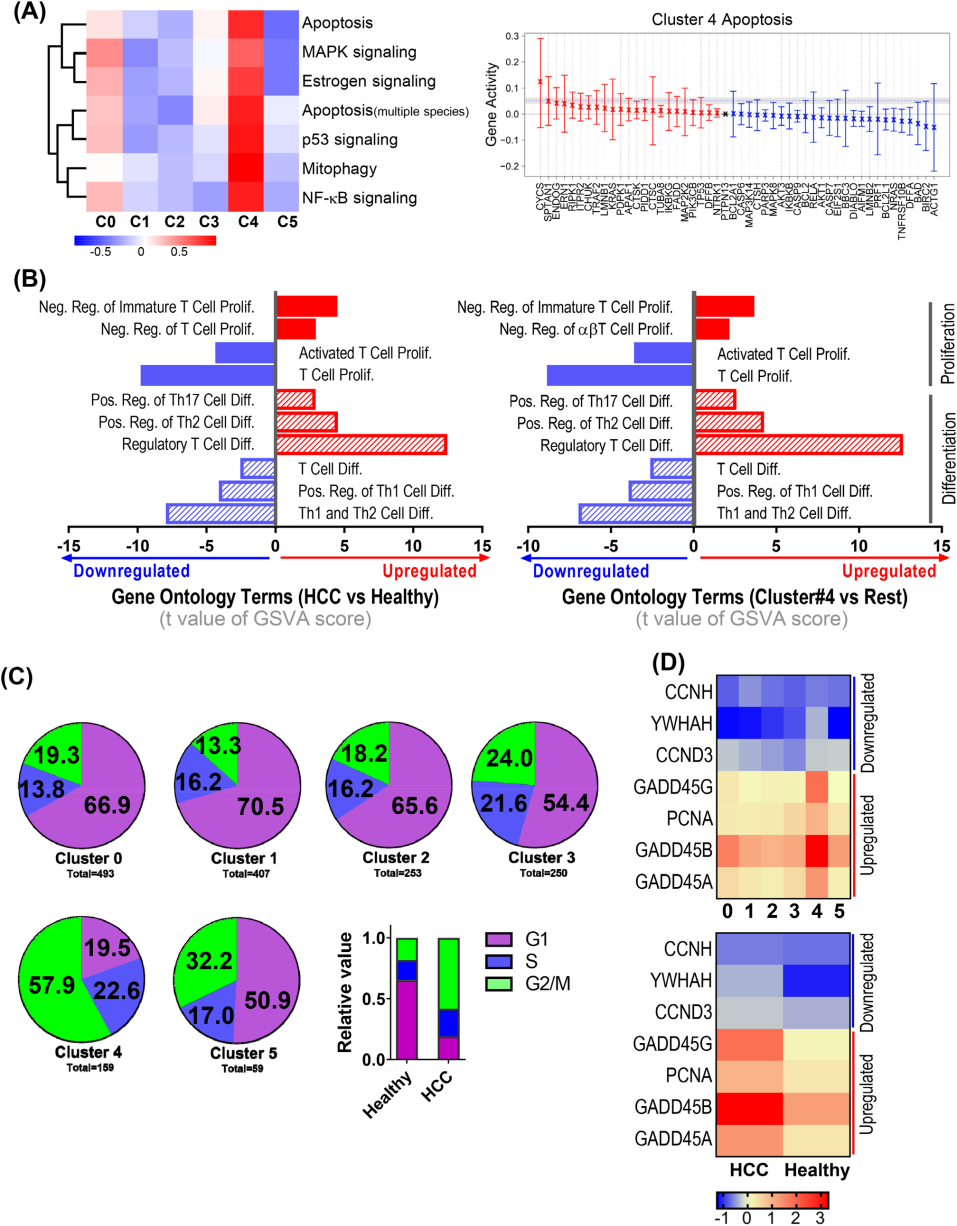
Proliferation, apoptosis and cell cycle‐related gene expression patterns in each cluster. (A) Heatmap analyses showing various apoptosis‐related pathway activities of individual clusters. (B) Gene ontology analyses of T cell proliferation, activation, differentiation and enrichment status of γδ T cells in hepatocellular carcinoma (HCC) versus healthy livers and γδ T cells in cluster 4 versus the rest of the clusters. (C) Pie chart showing the cell cycle stage of individual cells. (D) Expression patterns of cell cycle arrest and progression‐related genes of γδ T cells among individual clusters and between HCC and healthy livers

To understand whether the loss of diversity in HCC tumour‐infiltrated γδ T cells was due to limited T cell proliferation and/or induction of apoptosis in the TME, we compared apoptotic gene sets. Apoptosis‐related terms were more enriched in C4 than in the other clusters, implying that cells in C4 undergo active apoptosis (Figure 4A). Interestingly, the mitophagy pathway was also greatly upregulated in C4, correlating well with the suppressed OXPHOS metabolism in this cluster. We also performed gene set variation analysis (GSVA) of proliferation, differentiation and apoptosis‐related GO terms between HCC‐ and healthy liver‐infiltrating γδ T cells (Figure S4). We identified a loss of proliferation capacity and a shift in cellular fate to Th2, Th17 and regulatory T cell (Treg) differentiation (Figure 4B). We further analysed the cell cycle phases of individual cells in all clusters. Notably, nearly 60% of γδ T cells in C4 were in the G2/M phase. However, the γδ T cells in the remaining clusters were mainly in G1 (Figure 4C). Among the molecules that regulate this process, interaction with GADD45 family proteins potentially leads to G2/M arrest.^26^ Cell cycle‐related gene expression in HCC indicated downregulation of positive cell cycle regulators such as *CCNH* and *CCND3* and upregulation of cell cycle arrest‐related genes such as those of the *GADD45* family and *PCNA* (Figure 4D). The observed G2/M arrest of tumour‐infiltrating γδ T cells was likely due to the immunosuppressive HCC TME, which might lead to DNA damage‐induced cell cycle arrest.

### 3.6 HCC‐infiltrating γδ T cells were cytotoxic but exhausted

**FIGURE 5:**
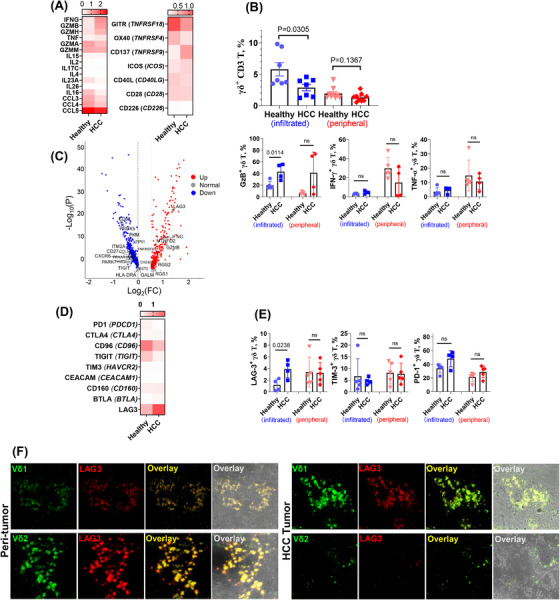
Cytotoxic molecules, cytokines and chemokine‐related gene expression and exhaustion gene profile of γδ T cells. (A) Gene expression heatmap of major cytokines, chemokines and T cell activation molecules of γδ T cells in hepatocellular carcinoma (HCC) versus healthy livers. (B) Flow cytometry analysis of functional molecule, cytokine and chemokine expression in γδ T cells from liver perfusates and peripheral blood mononuclear cells (PBMCs) of healthy donors and HCC+ patients (*n* = 5 per group). (C) Volcano map highlighting the most up‐ and downregulated genes related to T cell functions in HCC‐infiltrating γδ T cells. (D) Heatmap showing main immune inhibitory gene expression patterns of γδ T cells in HCC versus healthy livers. (E) Flow cytometry analysis of inhibitory molecule expression in γδ T cells from liver perfusates and PBMCs of healthy donors and HCC patients (*n* = 5 per group). (F) Confocal images of HCC tumour and peri‐tumour tissue‐infiltrating Vδ1^+^ and Vδ2^+^ γδ T cells and corresponding LAG3 expression (63X OIL). ns, no significance

We further evaluated the expression of both cytotoxic and inhibitory molecules at the transcriptional and protein levels. Various cytotoxic molecules and cytokine genes were expressed in γδ T cells from HCC and healthy samples (Figure 5A). Flow cytometry was then used to analyse both peripheral and infiltrating γδ T cells (Figure 5B), demonstrating that the HCC liver had a significantly lower number of γδ T cells than the healthy liver, although the proportion of γδ T cells was similar in the peripheral blood of healthy donors and HCC patients. Moreover, functional phenotyping (Figure 5B) exhibited no significant differences in the levels of cytotoxic molecules (IFN‐γ, TNF‐α) in peripheral and infiltrating γδ T cells, except for a significant increase in granzyme B in HCC‐infiltrating samples (*p* = .0114), which supports our transcriptomics data. We further found that the expression of LAG3, but not other checkpoint molecules, was highly upregulated both at the transcript and protein levels in HCC‐infiltrating γδ T cells (Figure 5C–E). Moreover, confocal fluorescence imaging of HCC tumour and peri‐tumour tissues (Figure 5F) indicated that all γδ T cells (Vδ1^+^ and Vδ2^+^) sturdily express LAG3, whereas PD1 was almost undetectable (Figure S5). In addition, Vδ2^+^ γδ T cells were less numerous than Vδ1^+^ γδ T cells in the TME. Notably, LAG3 protein expression was not upregulated in peripheral HCC γδ T cells compared with that in healthy controls (Figure 5C–E). This implies a LAG3‐mediated inhibition and exhaustion mechanism of infiltrating γδ T cells in the HCC TME.

### 3.7 HCC‐infiltrating γδ T cells exhibited profound alterations in metabolism

**FIGURE 6:**
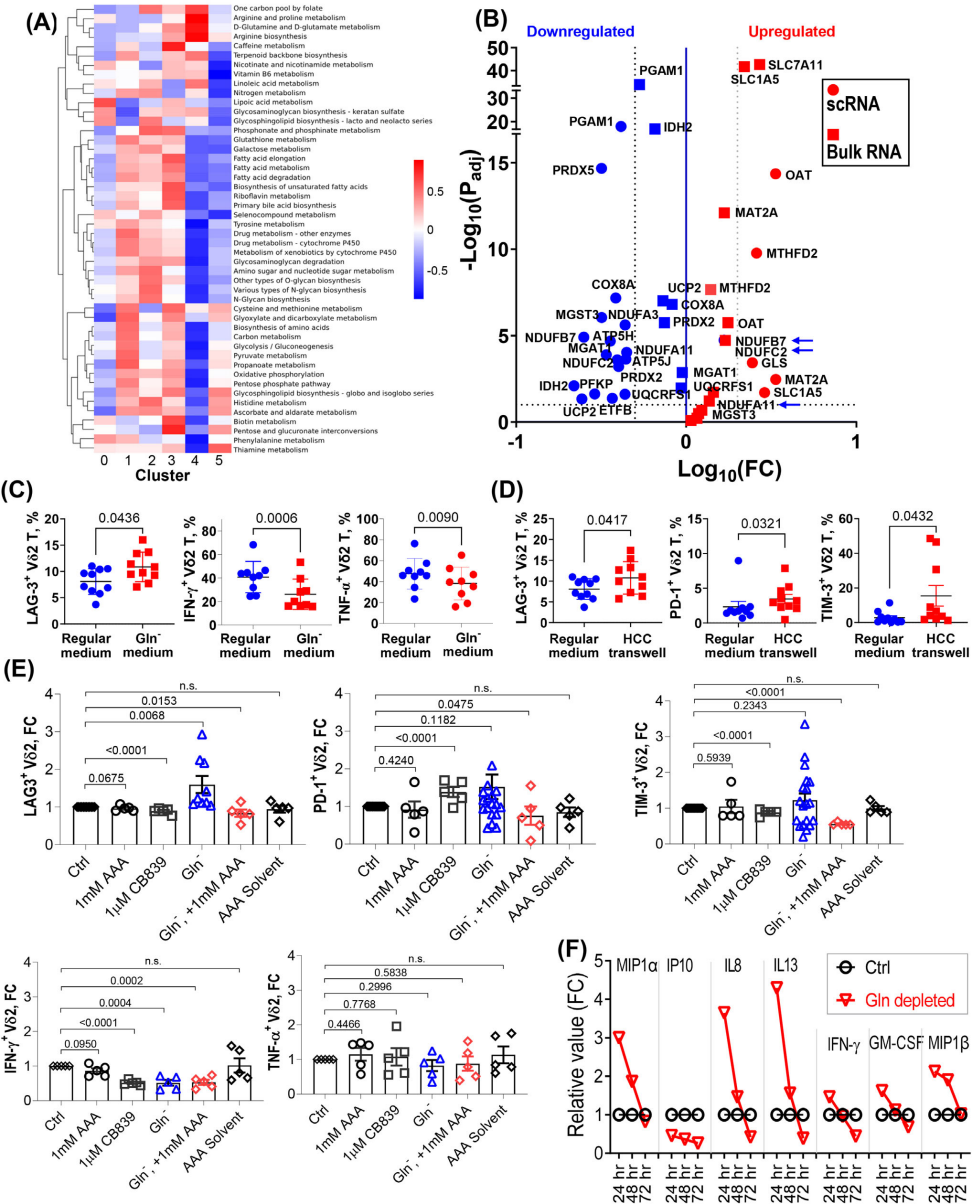
Metabolic pathways are altered in hepatocellular carcinoma tumour‐infiltrating γδ T cells (C4) comparing with clusters (C0–3, 5) of healthy liver‐resident γδ T cells. (A) Heatmap of individual metabolic pathways being regulated among clusters. (B) Volcano plot of up‐ or downregulated metabolic‐related genes between γδ T cells in hepatocellular carcinoma (HCC+) tumours and healthy livers. The round dots represent results obtained from single‐cell RNA sequencing of HCC‐infiltrating γδ T cells, and the square dots represent results obtained from bulk RNA sequencing of γδ T cells after 72‐h glutamine‐deficient culture conditions. (C) Flow cytometry analysis of inhibitory molecules: LAG3, PD‐1 and TIM3 expression of γδ T cells under regular and glutamine‐deficient culture conditions. A similar analysis was also performed for cytokines and cytotoxic molecules: IFN‐γ, TNF‐α, granzyme B and CD107a (*n* = 10 per group). (D) Flow cytometry analysis of inhibitory molecules: LAG3, PD‐1 and TIM3 expression of γδ T cells under regular and HCC transwell culture conditions. A similar analysis was also performed for cytokines and cytotoxic molecules: IFN‐γ, TNF‐α, granzyme B and CD107a (*n* = 10 per group). (E) Flow cytometry analysis of inhibitor and cytokine expression of γδ T cells under glutamine synthesis inhibition by D,L‐2‐aminoadipic acid (AAA) (1 mM), glutamine metabolism inhibition by CB839 (1 μM), glutamine‐deficient culture medium coupled with AAA and glutamine‐deficient culture medium conditions (*n* = 5 per group). (F) Cytokines and chemokines released from γδ T cells under 24‐, 48‐ and 72‐h time points under glutamine‐deficient culture conditions evaluated by Luminex 34‐plex cytokine/chemokine assay (*n* = 3). ns, no significance

We further focused on alterations in the major metabolic pathways of γδ T cells in the HCC TME. Effector T cell function‐correlated pathways such as glycolysis, OXPHOS, and fatty and amino acid metabolism were significantly downregulated, whereas glutamine metabolism and related pathways such as nitrogen, arginine and proline metabolism were upregulated in HCC‐infiltrating γδ T cells (Figure 6A, cluster 4). The expression of *SLC1A5*, a glutamine transporter, was significantly upregulated in the HCC‐infiltrating γδ T cell population. The expression of genes directly regulating glutamine metabolism, such as *OAT* and *GLS*, was also significantly upregulated. However, glycolysis‐related genes such as *PGAM1* and OXPHOS‐related genes such as *COX8A*, the *NDUF* family, and adenosine triphosphate (ATP) synthesis‐related genes *ATP5H* and *ATP5J* were all significantly downregulated. Bulk RNA sequencing identified a strong correlation in key metabolic gene expression patterns of ex vivo‐expanded γδ T cells from healthy donors in glutamine‐rich or ‐deficient conditions with cells from HCC or healthy livers (Figure 6B).

### 3.8 HCC‐infiltrating γδ T cells showed LAG3‐dependent dysfunction

Immunosuppressive gene expression is involved in regulating T cell metabolism. The overexpression of *LAG3* in HCC‐infiltrating γδ T cells might contribute to the suppressed effector T cell metabolic phenotype. Flow cytometry was used to evaluate the impact of the glutamine supply on the effector functions of ex vivo‐expanded Vδ2^+^ T cells (Figure 6C–F). Expression of LAG3, but not PD‐1 or TIM‐3, was significantly elevated in γδ T cells under extracellular glutamine restriction. Moreover, decreased levels of IFN‐γ and TNF‐α, rather than granzyme B and CD107a, were observed in the glutamine‐deficient medium (Figures 6C and S6A). Moreover, flow cytometry demonstrated that the expression of LAG3 was also upregulated under transwell co‐culture conditions, consistent with the scRNA‐seq results (Figure 6D). In the transwell, expression levels of two other inhibitory molecules, PD1 and TIM‐3, were also elevated. However, the levels of soluble molecules (IFN‐γ, TNF‐α, granzyme B and CD107a) released by Vδ2^+^ T cells showed no significant differences (Figures 6D and S6B).

We further used two glutamine metabolism inhibitors, AAA and CB839, to inhibit glutamine synthetase (GS) and glutaminase (GLS1) activity, respectively. Flow cytometry demonstrated that AAA, which restricts overall glutamine synthesis in γδ T cells, reversed the *LAG3* expression phenotype in glutamine‐deficient medium; blocking overall glutamine catabolism with CB839 showed a similar phenotype. This implies that the upregulation of *LAG3* expression under extracellular glutamine restriction was due to upregulation of the intracellular glutamine metabolic pathway. A similar result was observed with TIM‐3, but not with PD‐1 (Figure 6E).

Luminex 34‐plex cytokine/chemokine assays showed that under extracellular glutamine deprivation, levels of proinflammatory cytokines, including IFN‐γ, MIP1a, MIP1b, IL‐8, IL‐13 and GM‐CSF, were increased at 24 h while dropped significantly at 72 h (Figure 6F). No other molecules showed significant changes in production (Figure S6C). These data emphasise that prolonged exposure of γδ T cells to the glutamine‐deficient TME might impair their ability to secrete proinflammatory cytokines.

### 3.9 Ex vivo‐expanded Vδ2^+^ γδ T cells complement loss of TCR diversity and effector function of HCC‐infiltrating γδ T cells

**FIGURE 7:**
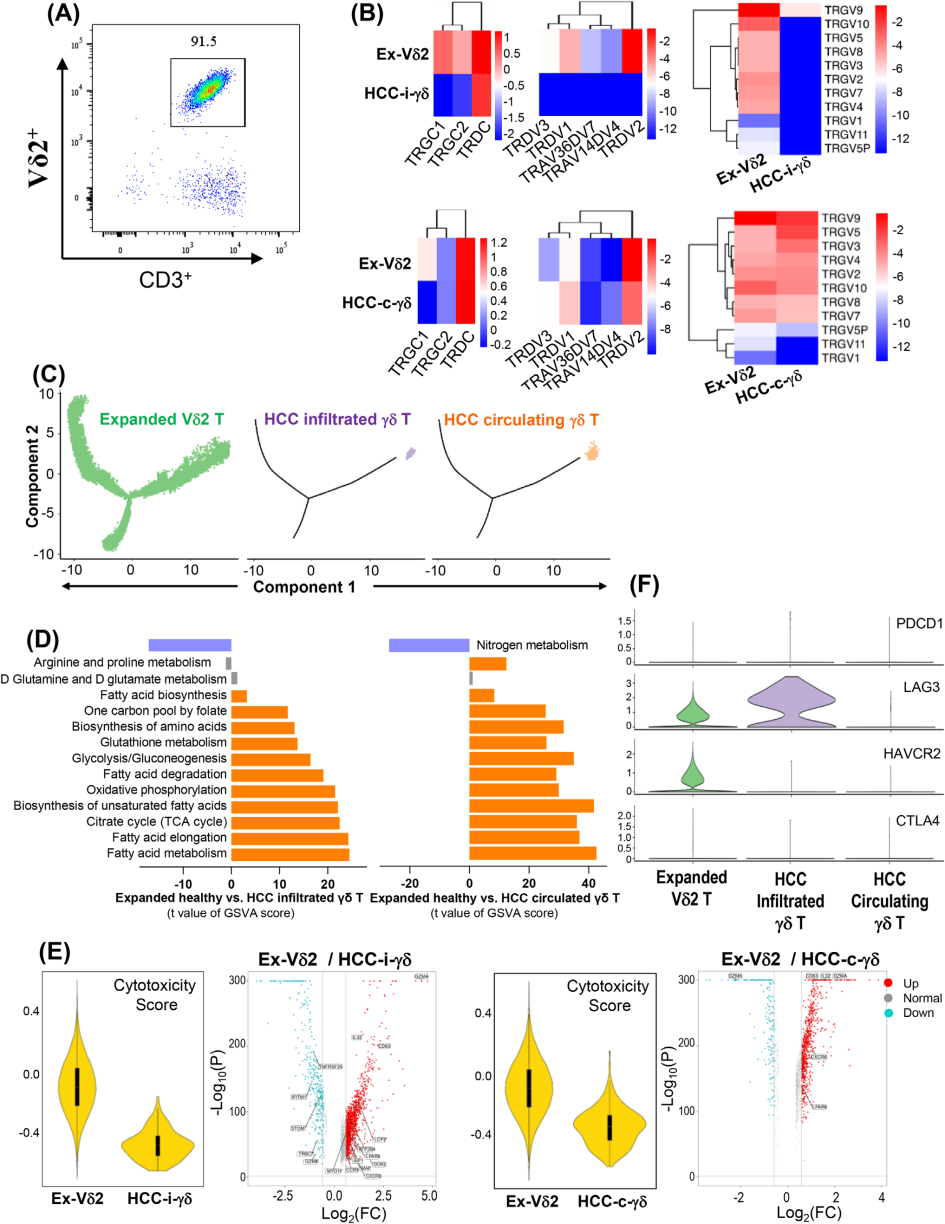
Ex vivo‐expanded γδ2+ T cells could rescue the functional and metabolic loss in hepatocellular carcinoma‐infiltrating γδ T cells. (A) Flow cytometry showed ex vivo‐expanded γδ2+ T cells from healthy donors achieved over 90% purity in the overall CD3 population. (B) Heatmap showing that ex vivo‐expanded γδ2+ T cells could complement the loss of T cell receptors in hepatocellular carcinoma (HCC)‐infiltrating γδ T cells. However, no discernible difference was observed between ex vivo‐expanded and peripheral γδ2+ T cells from healthy donors and HCC patients, respectively. (C) Pseudo‐time profile of ex vivo‐expanded, HCC‐infiltrating, and HCC peripheral γδ T cells. (D) Gene set variation analysis enrichment of main metabolic pathways in ex vivo‐expanded versus HCC‐infiltrating γδ T cells and ex vivo‐expanded versus HCC peripheral γδ T cells. (D) Cytotoxicity score and corresponding cytotoxic gene set volcano map of ex vivo‐expanded versus HCC‐infiltrating γδ T cells and ex vivo‐expanded versus HCC peripheral γδ T cells. (E) Violin plot showing the inhibitory gene expression patterns in ex vivo‐expanded, HCC‐infiltrating and HCC peripheral γδ T cells, respectively

We previously demonstrated that Vδ2^+^ γδ T cells expanded from healthy donor PBMCs were promising for the treatment of late‐stage cancer, including HCC.^12^, ^13^ We observed that infused Vδ2 T cells could rapidly migrate to and accumulate at tumour sites. Therefore, it is of interest whether ex vivo‐expanded Vδ2 T cells could complement the loss of the TCR diversity and effector functionality of the HCC‐infiltrating γδ T cells. To address this question, we conducted scRNA‐seq profiling of expanded Vδ2 T cells from PBMCs of three healthy donors (Figure 7A). The t‐distributed stochastic neighbourhood embedding (t‐SNE) analysis determined that there was no significant heterogeneity among expanded Vδ2 T cells from the three different healthy donors (Figure S7A). We found higher enrichment of major γδ TCRs in the expanded cells than in infiltrating (but not peripheral) HCC‐derived cells (Figure 7B). A differentiation trajectory demonstrated a branched structure in expanded Vδ2 T cells, implying functional and differentiation diversity. However, this observation was lost in HCC samples (Figure 7C). GSVA of metabolic pathways demonstrated enrichment of major metabolic pathways such as OXPHOS, glycolysis and fatty acid metabolism in expanded Vδ2 T cells versus HCC‐derived cells (Figure 7D). Further GSVA comparisons of cytotoxic genes showed a higher cytotoxicity score for expanded Vδ2 T cells than for HCC samples (Figure 7E), implying potential complementation of lost cytotoxic effector function. Finally, gene expression levels showed that *LAG3* was mainly expressed in infiltrating, but not peripheral, HCC‐derived γδ T cells (Figure 7F). As for γδ T cells in healthy donor PBMCs, a resting state was maintained with nearly no expression of inhibitory molecule‐related genes (Figure S7B,C). These results suggest that ex vivo‐expanded Vδ2^+^ γδ T cells could indeed complement the loss of the anti‐tumour functionality of HCC‐infiltrating γδ T cells, also supporting the potential of ex vivo‐expanded Vδ2^+^ γδ T cells for the treatment of HCC.

## 4 DISCUSSION

HCC is one of the most difficult cancers to treat. Immunotherapy has increasingly become one of the most promising treatment strategies for solid tumours; however, only limited responses in isolated HCC cases have been reported. PD‐1 checkpoint blockade led to tumour regression in ∼20% of patients with advanced HCC,^27^ and clinical trials on cytotoxic T‐lymphocyte‐associated antigen 4 (CTLA‐4) blockade showed a partial response in ∼26% of HCC cases.^28^ Although there are a few ongoing trials focused on LAG3 (clinicaltrials.gov), none have shown definitive efficacy thus far. The relatively low response rate to checkpoint blockade treatments might be partly due to the heterogeneous nature of the HCC TME. Furthermore, switching from an OXPHOS‐favouring environment to an acidic glycolytic environment might influence the expression of checkpoint molecules in infiltrating lymphocytes.^29^ Due to the multifaceted and immunosuppressive nature of the TME, the balance between pro‐ and anti‐tumour effects of tumour‐infiltrating lymphocytes (TILs) often skews towards a tumour‐supporting environment. Specifically, γδ T cells are often exhausted or impaired.^30^ Nevertheless, γδ T cell plasticity allows for differentiation into pro‐ or anti‐tumour subsets.^17^, ^31^

scRNA‐seq provides insight into the lineage and functionalities of TILs.^32^ HCC tumours harbour many immune cells, which contribute to clinical prognosis. Nevertheless, the immune signatures of γδ T cells are not fully understood at the single‐cell level, particularly in the context of the HCC TME. Accordingly, the aim of this study was to understand the effector functions and metabolic alterations of γδ T cells in HCC patients. We identified a suite of prominent variations between infiltrating γδ T cells in HCC and healthy liver tissues. We hypothesise that, although tumour‐recruited γδ T cells retain cytotoxicity against cancer cells, this is eroded over time by chronic HCC challenge in the TME. Specifically, HCC‐infiltrating γδ T cells displayed cytotoxic but exhausted gene expression patterns, as evidenced by the overexpression of both cytotoxicity‐related and exhaustion/inhibitory genes. The upregulated mitophagy pathway supported this explanation. Mitophagy is a key mitochondrial quality control mechanism; the remarkable activation of mitophagy of HCC‐infiltrating γδ T cells indicates active rebuilding of cellular homeostasis in response to TME stress. Moreover, metabolic pathways such as OXPHOS, glycolysis and fatty and amino acid biosynthesis were significantly suppressed and accompanied by downregulation of cytoskeletal, adhesion and leucocyte trans‐endothelial migration‐related genes, leading to a transition to glutamine metabolism to maintain minimal cellular activity.

TCR clonality and the T cell differentiation trajectory are often used to understand the evolution and functional variations of different T cell sub‐clusters. We found a drastic loss of γδ T cell TCR diversity in HCC tumours, which might be due to activation‐induced apoptosis and exhaustion. In humans, the two major γδ T cell subtypes are characterised by the δ chain. The Vδ1 population mainly resides in the intestine, whereas the Vδ2 population predominates in the peripheral blood. Peripheral γδ T cells with Vδ1 or Vδ2 TCR displayed shared but distinct cytotoxic hallmarks and had similar differentiation trajectories. The Vδ1 subtype was observed as the main population in both tumour and peri‐tumour tissues. However, a reduced Vδ2 was found in HCC tissues, which can be interpreted as either impaired infiltration capability or selective depletion triggered by the long‐term stimulation of phosphate‐associated antigens in the HCC TME. Vδ1 cells, especially those derived from tumours, are considered to suppress the tumour immune response.^17^, ^33^, ^34^ Therefore, analysing the TCR clonality of infiltrating γδ T cells from healthy and HCC liver tissues might help to illustrate this shift. Although scRNA‐seq did not produce meaningful Vδ1 or Vδ2 expression profiles, flow cytometry indicated a shift in the peripheral blood of HCC patients towards a higher Vδ1 percentage in the overall γδ T cell population. The ratios of both cytotoxic and regulatory subgroups, but not the inflammatory subgroup of γδ T cells, decreased in the peripheral blood of HCC patients, implying a loss of effector functions.

Additionally, metabolic transcriptomic profiles of HCC‐infiltrating γδ T cells indicated downregulation of metabolic pathways such as glycolysis and OXPHOS, which are necessary for effective anti‐tumour functions. The question of how specific metabolic pathways modulate the differentiation and functions of T cell subsets has yet to be addressed; the answer may allow for the selective modulation of T cell immunity within tumours. One study indicated plasticity in T cell metabolism that could be exploited for cancer immunotherapy.^35^ Our scRNA‐seq data showed upregulation of glutamine metabolism and downregulation of key effector T cell‐dependent metabolic pathways such as glycolysis and OXPHOS in HCC‐infiltrating γδ T cells. Glutamine is an important fuel source for both cancer and tumour‐infiltrating immune cells,^36^, ^37^ its metabolism is closely tied to nitrogen metabolism and provides most of the nitrogen required for nucleic acid and amino acid synthesis. Glutamine is critical for T cell activation, proliferation and differentiation, and low glutamine levels in the TME suppress effector T cell functions.^38^ Glutamine also regulates mammalian target of rapamycin (mTOR) activation^39^ and O‐glcNAcylation^40^ in effector T cells, two key steps for proper T cell development and function. Our in vitro results suggest that extracellular glutamine deprivation of γδ T cells decreased the levels of key effector cytokines such as IFN‐γ and TNF‐α, whereas cytotoxic molecules such as granzyme B and CD107a were unaffected. Thus, further studies are needed to explore whether limiting glutamine metabolism could strengthen effector γδ T cell anti‐tumour function in the TME.

Interestingly, LAG3 was the only known immune inhibitory gene that showed distinct upregulation at both the mRNA and protein expression levels in HCC‐derived γδ T cells. Our in vitro data imply that glutamine deficiency in the TME would be a key inducer of LAG3 upregulation. Unexpectedly, the transwell experiment showed that LAG3, PD1 and TIM3 were all upregulated. This is likely due to the miscellaneous molecules secreted by HCC cells in the transwell. Nevertheless, for HCC immunotherapy, LAG3 blockade would be more promising than blockade of PD1 and other checkpoint molecules as an adjuvant combination with the adoptive transfer of γδ T cells. Further mouse and clinical trials are needed to validate this. LAG3 inhibits the activity of CD4^+^ T cells,^41^ and the effector functions of CD8^+^ T cells and natural killer cells, raising the possibility of discovering new LAG3 ligands to manipulate LAG3‐mediated immune inhibitory functions.^42^ Fibrinogen‐like protein 1 (FGL1) is a major immune inhibitory ligand for LAG3.^41^ Moreover, galectin‐3,^43^ LSECtin^44^ and α‐synuclein^45^ interact with LAG3 and negatively regulate the anti‐tumour functions of T cells. FGL1 and LSECtin, both of which are overexpressed in healthy livers and downregulated in liver cancer patients according to The Cancer Genome Atlas, do not affect the long‐term survival of liver cancer patients. To investigate new potential ligands for LAG3 protein on γδ T cells, we also performed a pull‐down experiment using purified recombinant LAG3 protein, identifying 35–86 candidate ligands for LAG3, including MHC II proteins; further analysis is ongoing.

Compared with Vδ2^+^ γδ T cells, Vδ1^+^ γδ T cells predominantly exist in both the tumour tissue and peripheral blood of HCC patients. We thus propose the hypothesis that cytotoxic Vδ2^+^ γδ T cells are selectively depleted by chronic stimulation of phosphor‐related antigens of tumour cells through activation‐induced apoptosis (scRNA‐seq data revealed upregulation of apoptosis‐related genes). Therefore, transfer therapy of allogeneic Vδ2^+^ γδ T cells derived from healthy donors could rescue the functional defects of γδ T cells and re‐establish anti‐tumour immunity in cancer patients. We previously validated this hypothesis in clinical trials,^12^, ^13^ which unequivocally showed the efficacy of allogeneic Vδ2^+^ γδ T cells in late‐stage cancer patients. Moreover, infused Vδ2^+^ γδ T cells elevated the immune response of other immune cells such as CD8^+^ and CD4^+^ T cells and natural killer cells. Additionally, t‐SNE analysis of three groups of ex vivo‐expanded Vδ2^+^ T cells from three separate donors demonstrated high homogeneity in gene expression and clustering patterns as well as functional complements to HCC‐infiltrating γδ T cells. This demonstrated that (1) expanded Vδ2 T cells from different individuals are functionally homogeneous and can compensate for the functional deficiency of HCC‐infiltrating γδ T cells, and (2) allogeneic Vδ2 T cells can be developed into an off‐the‐shelf cell product as a basic vehicle for universal CAR‐T development.

Lastly, our work has a few limitations and thus further efforts are needed to illustrate the functional deficiency of HCC‐related γδ T cells. Specifically, rather than using a sample mixture for single‐cell sequencing, sequencing performed on respective samples could better illustrate the γδ T cell heterogeneity among patients. Meanwhile, increased number of sample cohort could further reduce potential bias caused from clinical stages of HCC, age, gender and other factors. Moreover, γδ T cells from HBV‐negative HCC patients should be analysed to address the possibility that the variation we observed in HBV‐related HCC patients could be due to chronic HBV infection. Additionally, since the number and function of γδ T cells are also reported to be sex related,^46^, ^47^, ^48^ enrolling patients of the same gender will be more desirable. Lastly, comparing the functional differences between Vδ 1 and Vδ 2 T cells in HCC will help understand the preferential enrichment of Vδ 1 in HCC TME.

In summary, we compared the metabolism, cytotoxicity, and TCR profiles of γδ T cells in HCC using scRNA‐seq. This work can complement the understanding of HCC‐infiltrating γδ T cells, which will facilitate the development of γδ T cell‐based immunotherapy. Our results support the hypothesis that ex vivo‐expanded Vδ2^+^ γδ T cells can functionally complement those in patients. Moreover, LAG3 might be a better therapeutic target than PD‐1 and CTLA‐4 for immune checkpoint therapy in HCC patients. Applying allogeneic γδ T cell transfer in combination with LAG3 checkpoint blockade should potentially maximise the anti‐tumour function of γδ T cells in HCC immunotherapy, which should be tested in future work.

## CONFLICT OF INTEREST

All authors declare no conflict of interest.

## Supporting information

Mateiral_S1A

Mateiral_S2A

Mateiral_S3A

Supporting information
