# Supplementary material for: Hepatocellular carcinoma‐infiltrating γδ T cells are functionally defected and allogenic Vδ2+ γδ T cell can be a promising complement
Source: Clin Transl Med. 2022 Apr 7;12(4):e800. doi: 10.1002/ctm2.800 (PMC8989380; doi:10.1002/ctm2.800)
Supplement: Supplementary file 1 — Mateiral_S1A [file CTM2-12-e800-s002.pptx]

## Slide 1
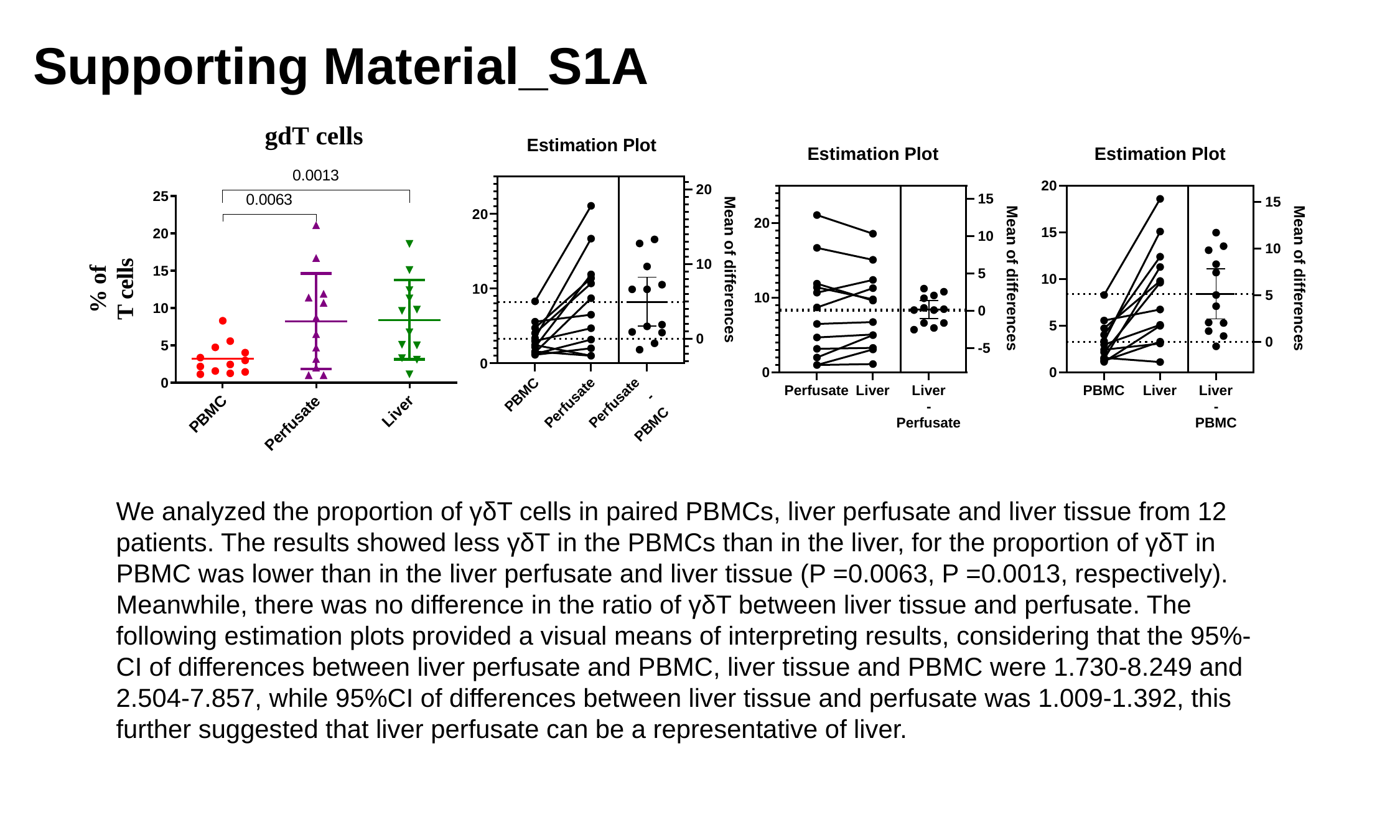

Supporting Material_S1A
We analyzed the proportion of γδT cells in paired PBMCs, liver perfusate and liver tissue from 12 patients. The results showed less γδT in the PBMCs than in the liver, for the proportion of γδT in PBMC was lower than in the liver perfusate and liver tissue (P =0.0063, P =0.0013, respectively). Meanwhile, there was no difference in the ratio of γδT between liver tissue and perfusate. The following estimation plots provided a visual means of interpreting results, considering that the 95%-CI of differences between liver perfusate and PBMC, liver tissue and PBMC were 1.730-8.249 and 2.504-7.857, while 95%CI of differences between liver tissue and perfusate was 1.009-1.392, this further suggested that liver perfusate can be a representative of liver.

## Slide 2
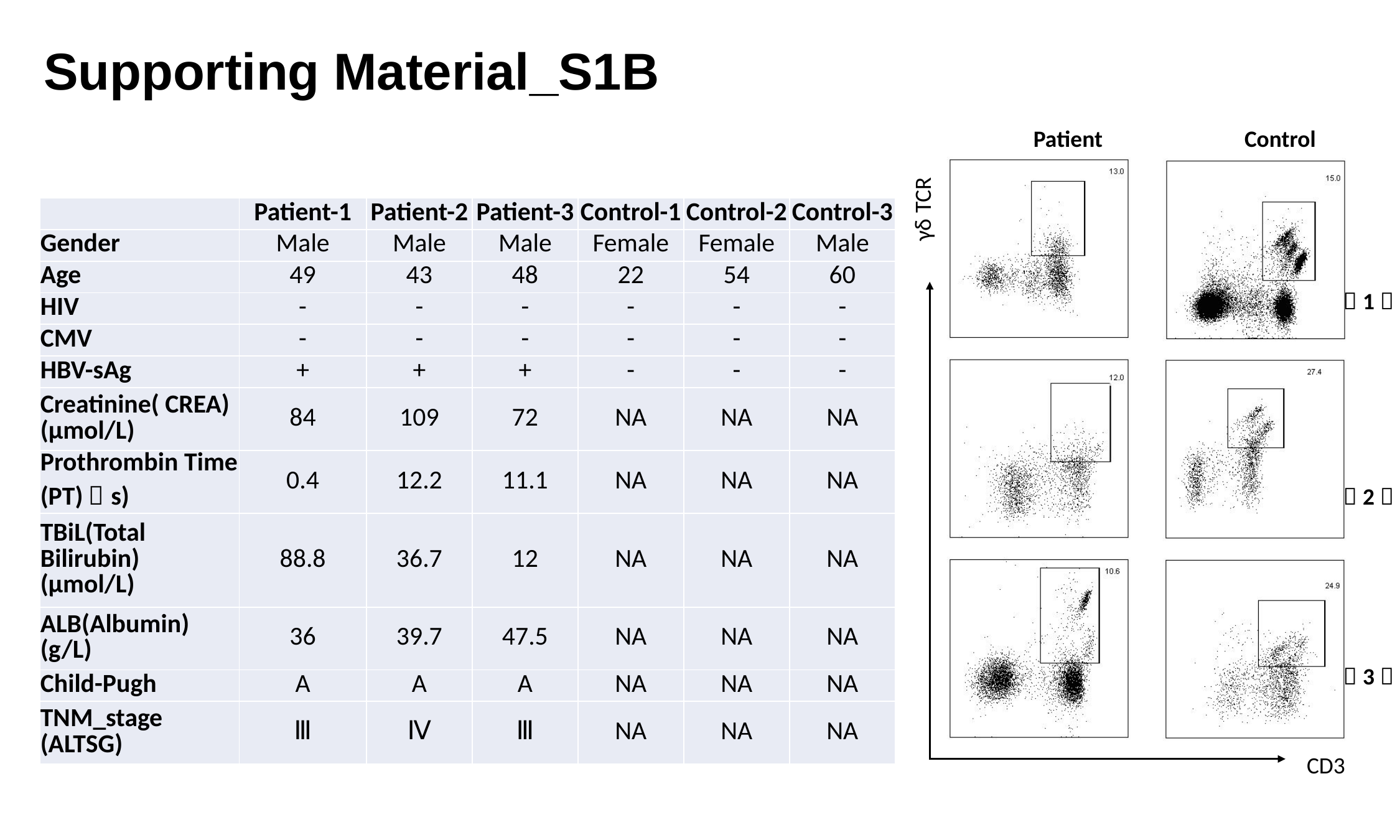

Supporting Material_S1B
Patient
Control
γδ TCR
CD3
| | Patient-1 | Patient-2 | Patient-3 | Control-1 | Control-2 | Control-3 |
| --- | --- | --- | --- | --- | --- | --- |
| Gender | Male | Male | Male | Female | Female | Male |
| Age | 49 | 43 | 48 | 22 | 54 | 60 |
| HIV | - | - | - | - | - | - |
| CMV | - | - | - | - | - | - |
| HBV-sAg | + | + | + | - | - | - |
| Creatinine( CREA) (μmol/L) | 84 | 109 | 72 | NA | NA | NA |
| Prothrombin Time (PT)（s) | 0.4 | 12.2 | 11.1 | NA | NA | NA |
| TBiL(Total Bilirubin) (μmol/L) | 88.8 | 36.7 | 12 | NA | NA | NA |
| ALB(Albumin) (g/L) | 36 | 39.7 | 47.5 | NA | NA | NA |
| Child-Pugh | A | A | A | NA | NA | NA |
| TNM\_stage (ALTSG) | Ⅲ | Ⅳ | Ⅲ | NA | NA | NA |
（1）
（2）
（3）
