# Supplementary material for: Hepatocellular carcinoma‐infiltrating γδ T cells are functionally defected and allogenic Vδ2+ γδ T cell can be a promising complement
Source: Clin Transl Med. 2022 Apr 7;12(4):e800. doi: 10.1002/ctm2.800 (PMC8989380; doi:10.1002/ctm2.800)
Supplement: Supplementary file 2 — Mateiral_S2A [file CTM2-12-e800-s001.pptx]

## Slide 1
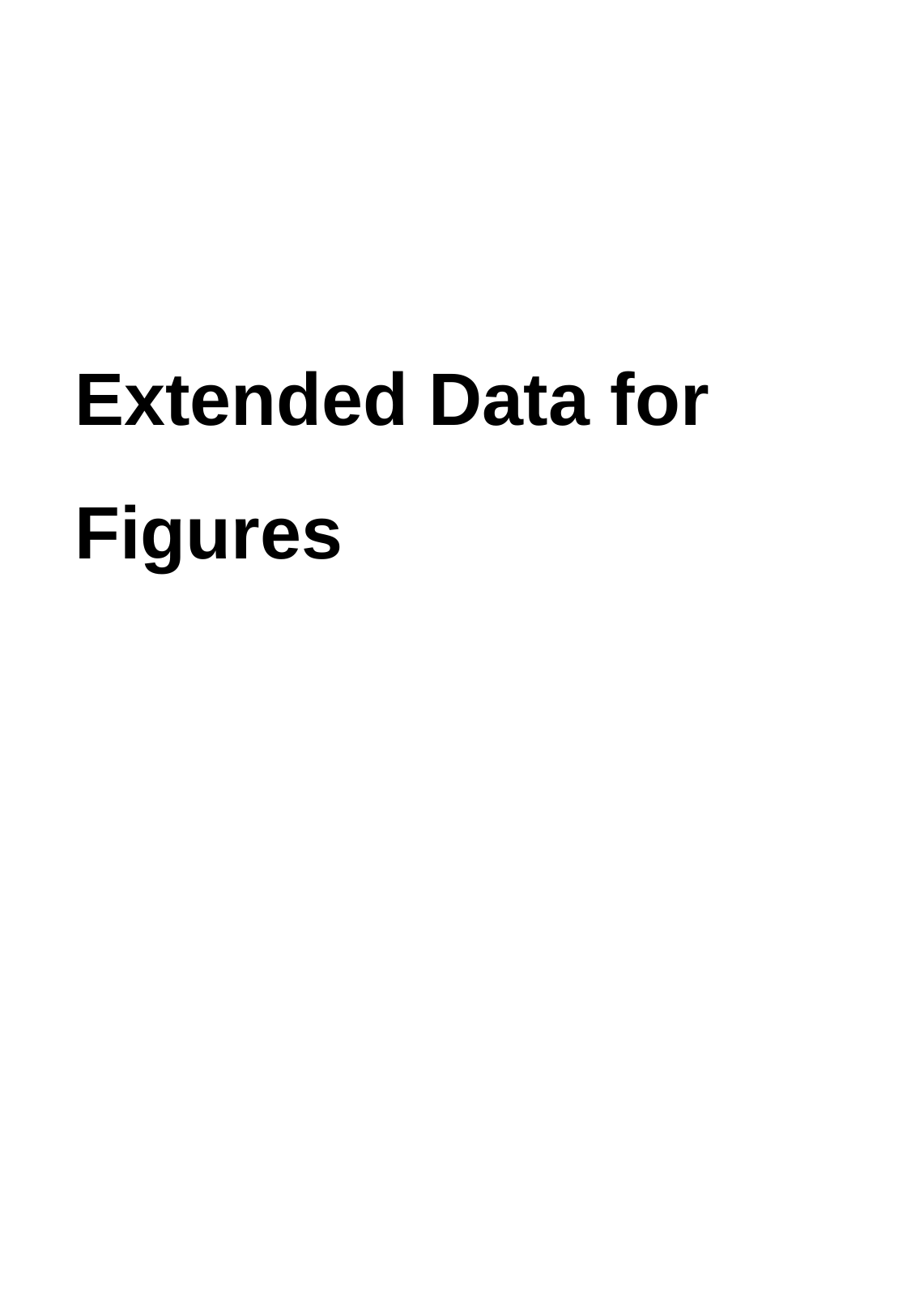

# Extended Data for Figures

## Slide 2
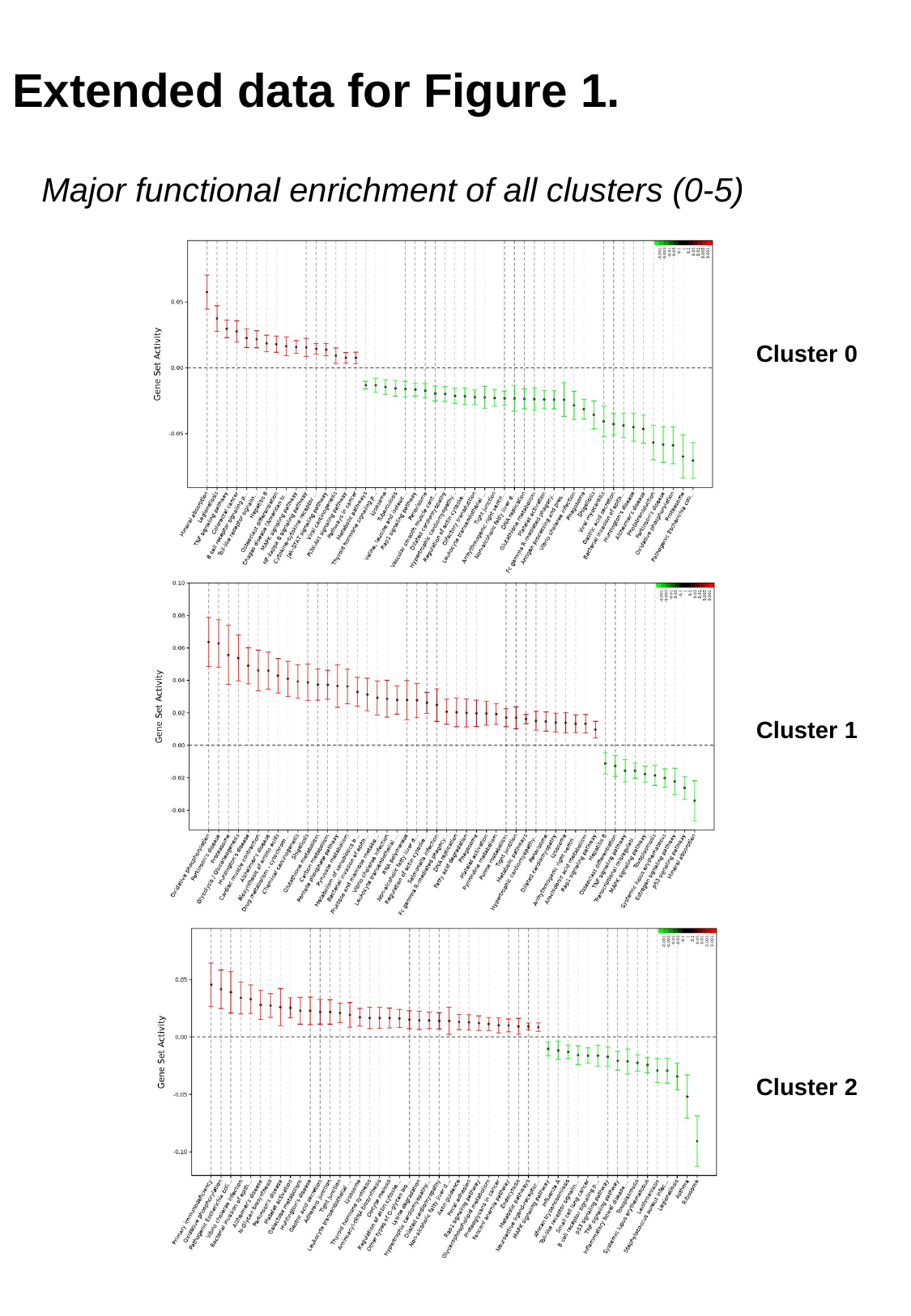

# Extended data for Figure 1.
Major functional enrichment of all clusters (0-5)
Cluster 0
Cluster 1
Cluster 2

## Slide 3
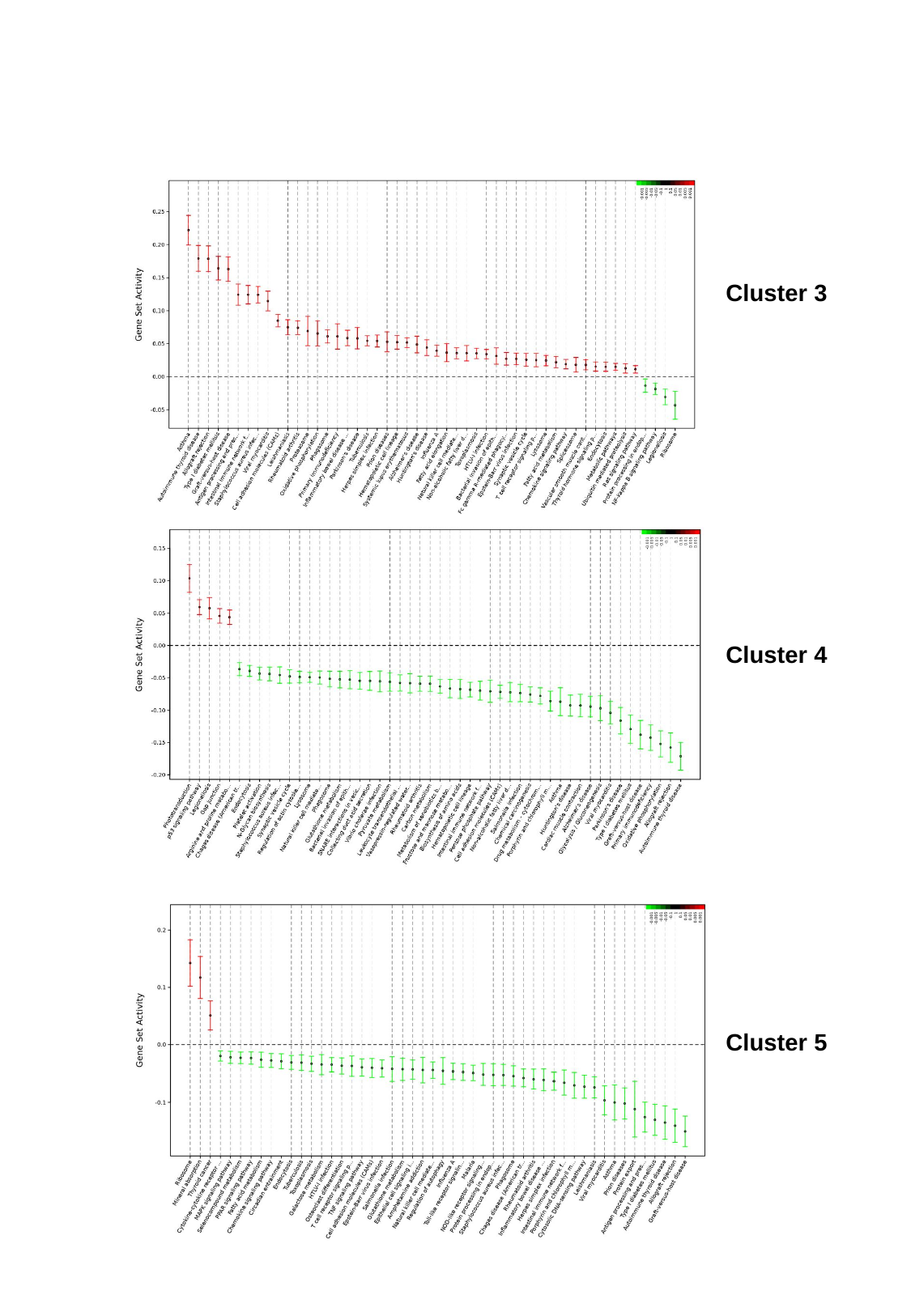

Cluster 3
Cluster 4
Cluster 5

## Slide 4
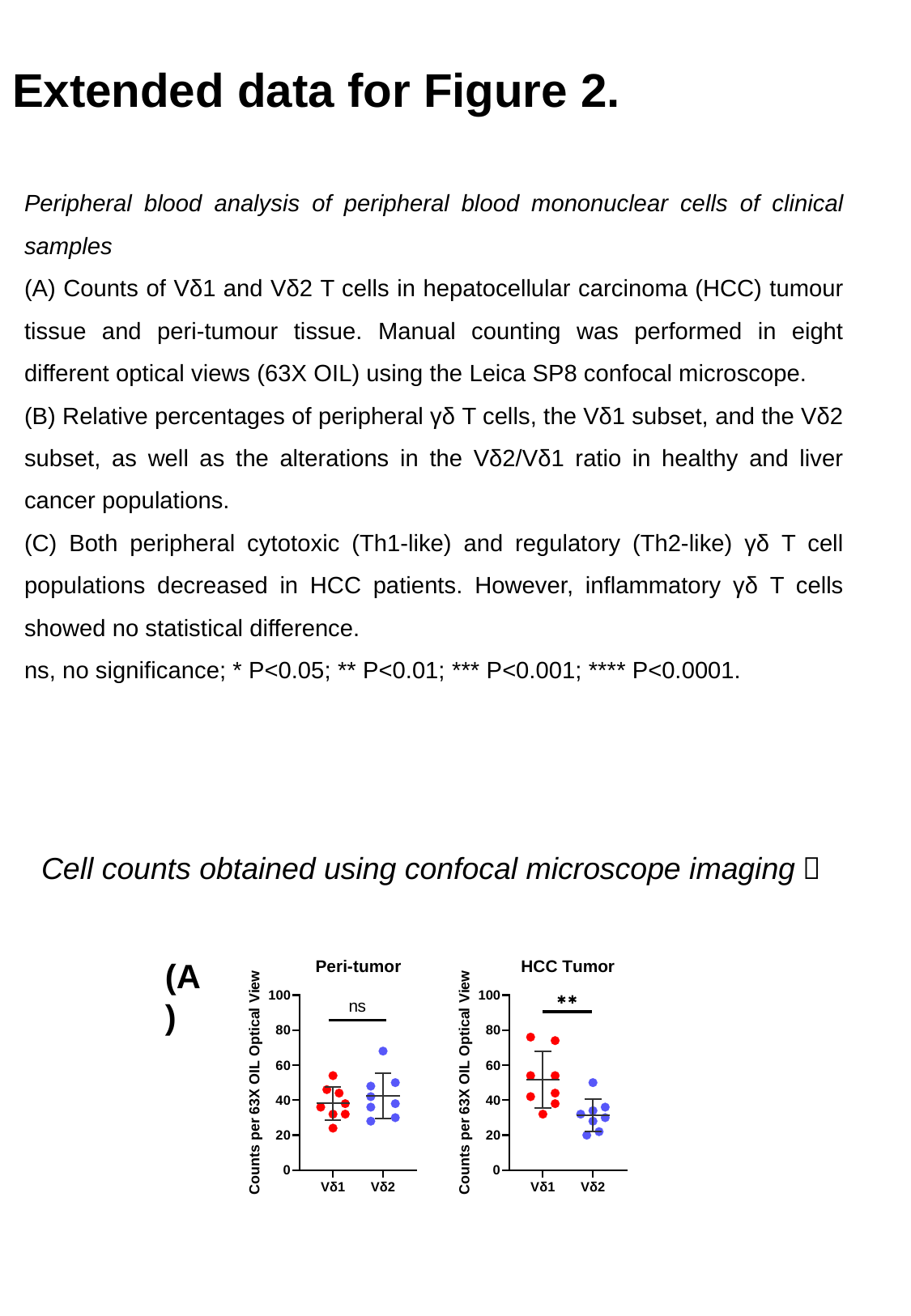

# Extended data for Figure 2.
Peripheral blood analysis of peripheral blood mononuclear cells of clinical samples
(A) Counts of Vδ1 and Vδ2 T cells in hepatocellular carcinoma (HCC) tumour tissue and peri-tumour tissue. Manual counting was performed in eight different optical views (63X OIL) using the Leica SP8 confocal microscope.
(B) Relative percentages of peripheral γδ T cells, the Vδ1 subset, and the Vδ2 subset, as well as the alterations in the Vδ2/Vδ1 ratio in healthy and liver cancer populations.
(C) Both peripheral cytotoxic (Th1-like) and regulatory (Th2-like) γδ T cell populations decreased in HCC patients. However, inflammatory γδ T cells showed no statistical difference.
ns, no significance; * P<0.05; ** P<0.01; *** P<0.001; **** P<0.0001.
Cell counts obtained using confocal microscope imaging：
(A)

## Slide 5
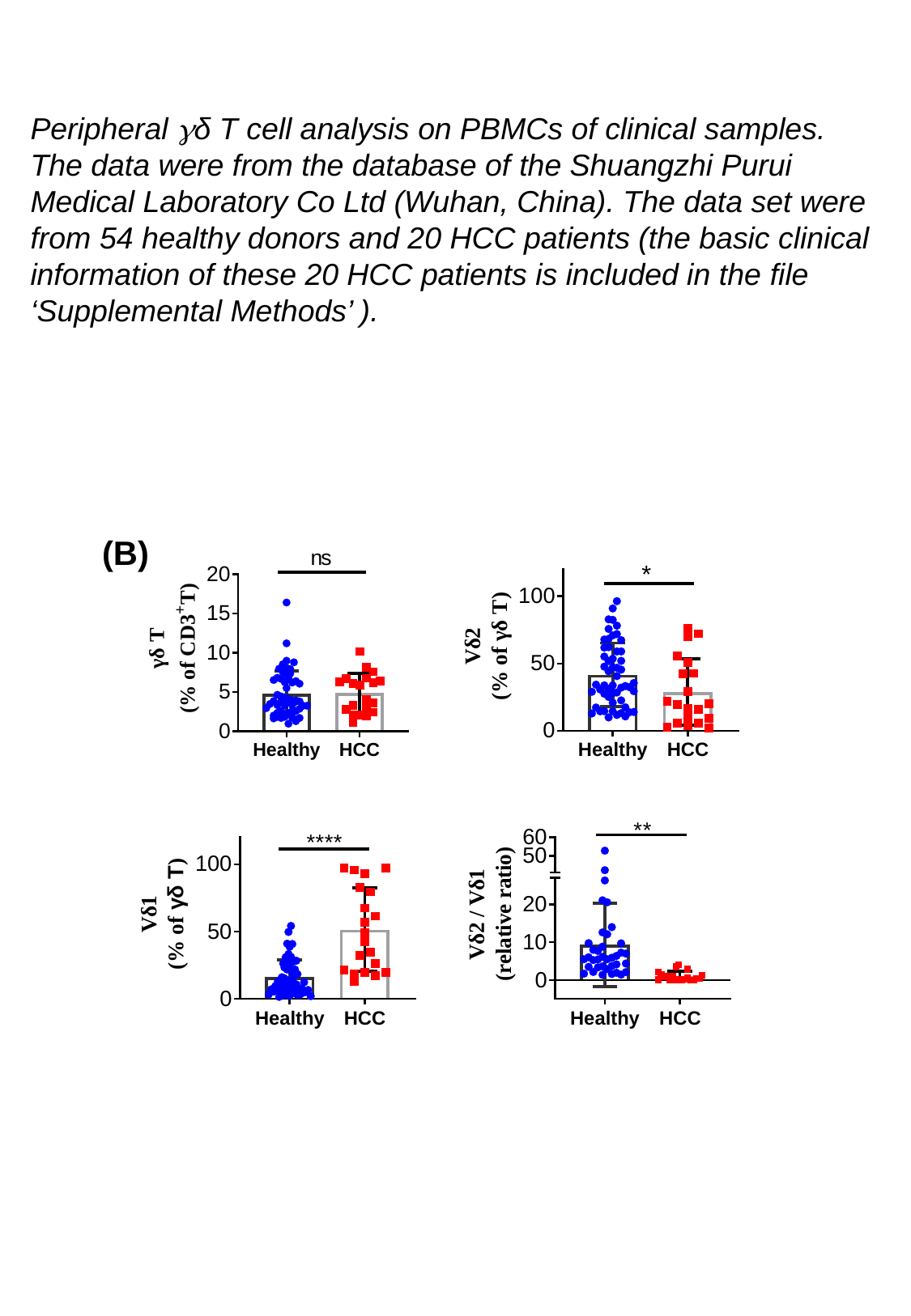

Peripheral δ T cell analysis on PBMCs of clinical samples.
The data were from the database of the Shuangzhi Purui Medical Laboratory Co Ltd (Wuhan, China). The data set were from 54 healthy donors and 20 HCC patients (the basic clinical information of these 20 HCC patients is included in the file ‘Supplemental Methods’ ).
(B)

## Slide 6
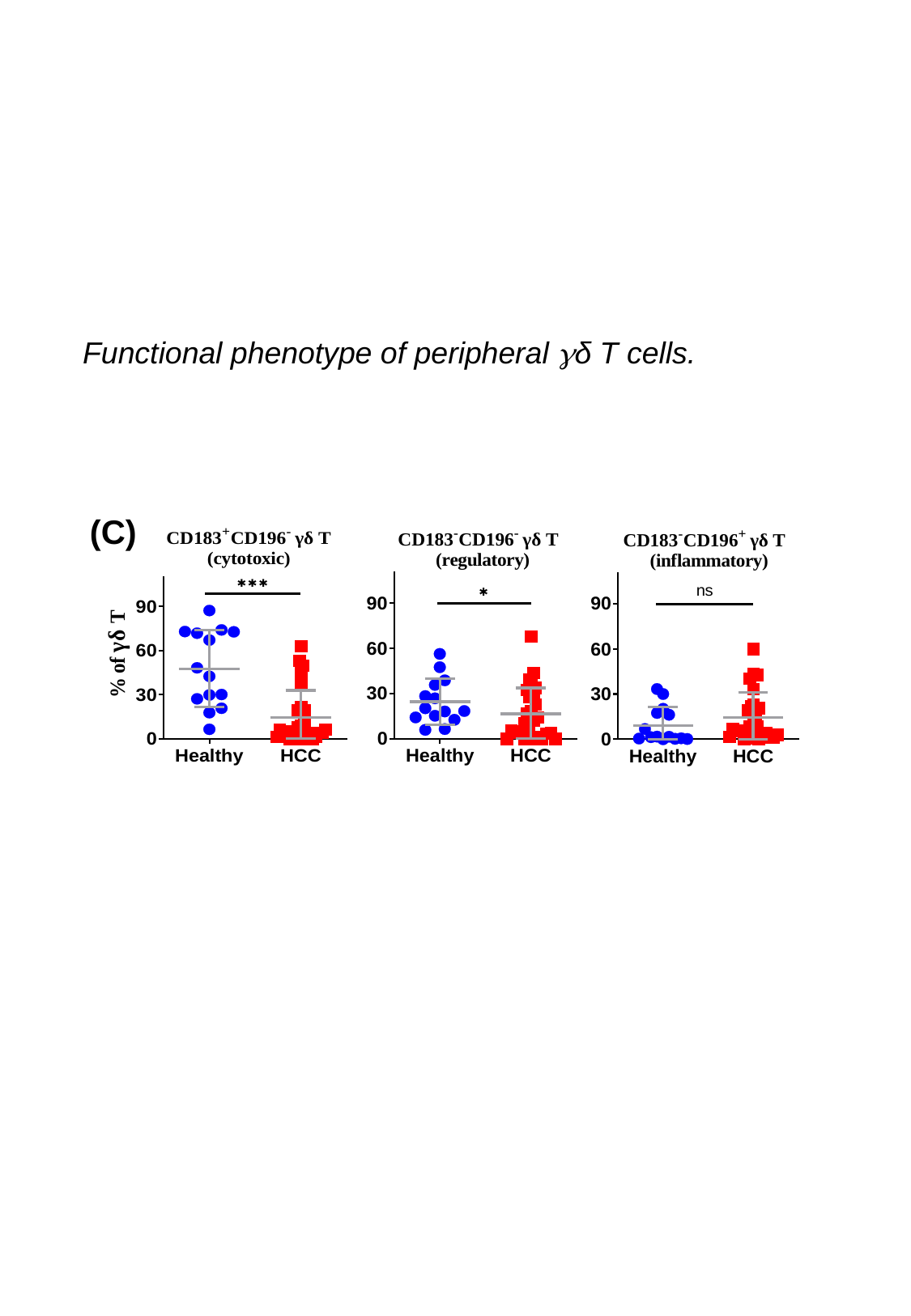

Functional phenotype of peripheral δ T cells.
(C)

## Slide 7
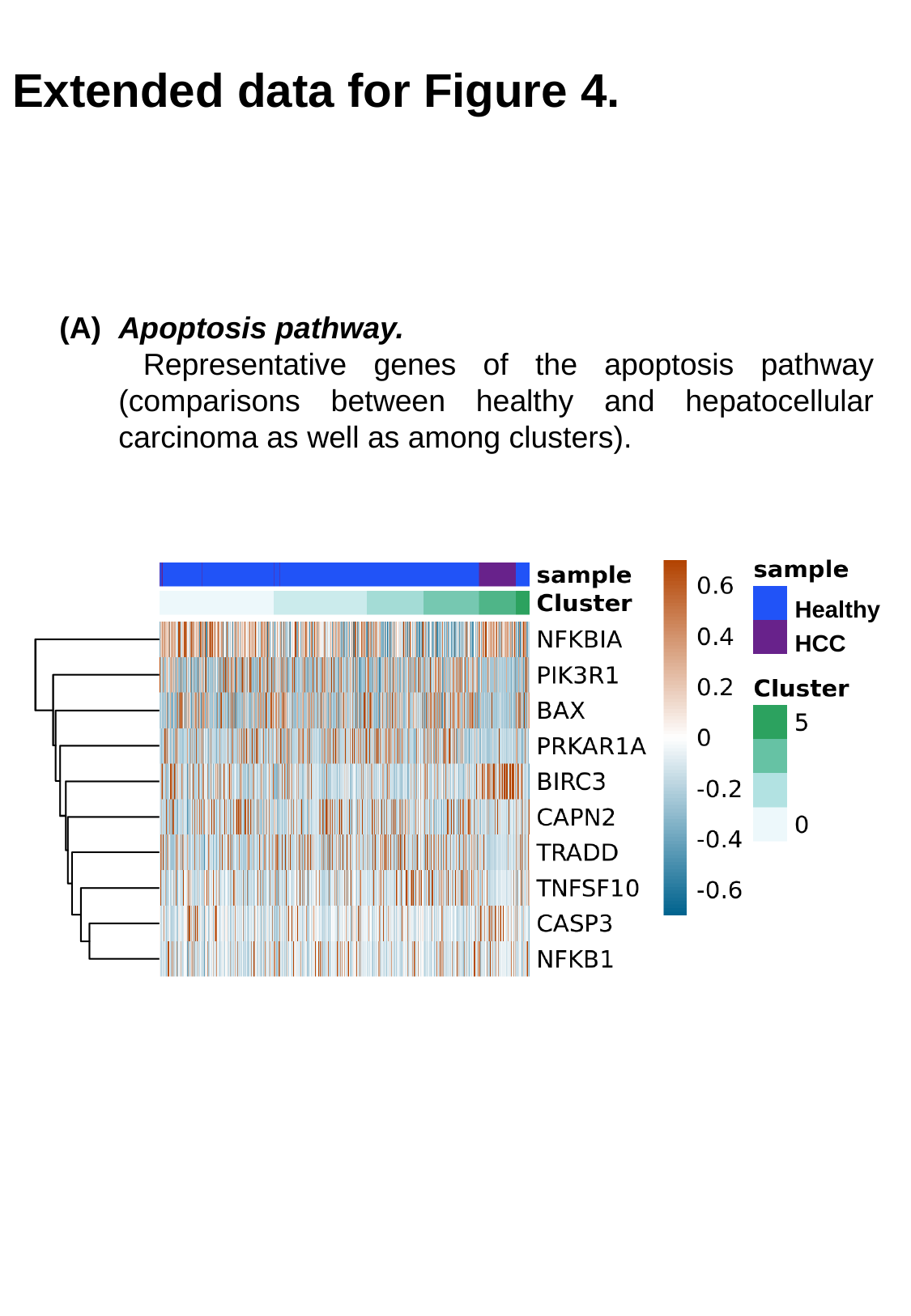

# Extended data for Figure 4.
(A) Apoptosis pathway.
 Representative genes of the apoptosis pathway (comparisons between healthy and hepatocellular carcinoma as well as among clusters).
Healthy
HCC

## Slide 8
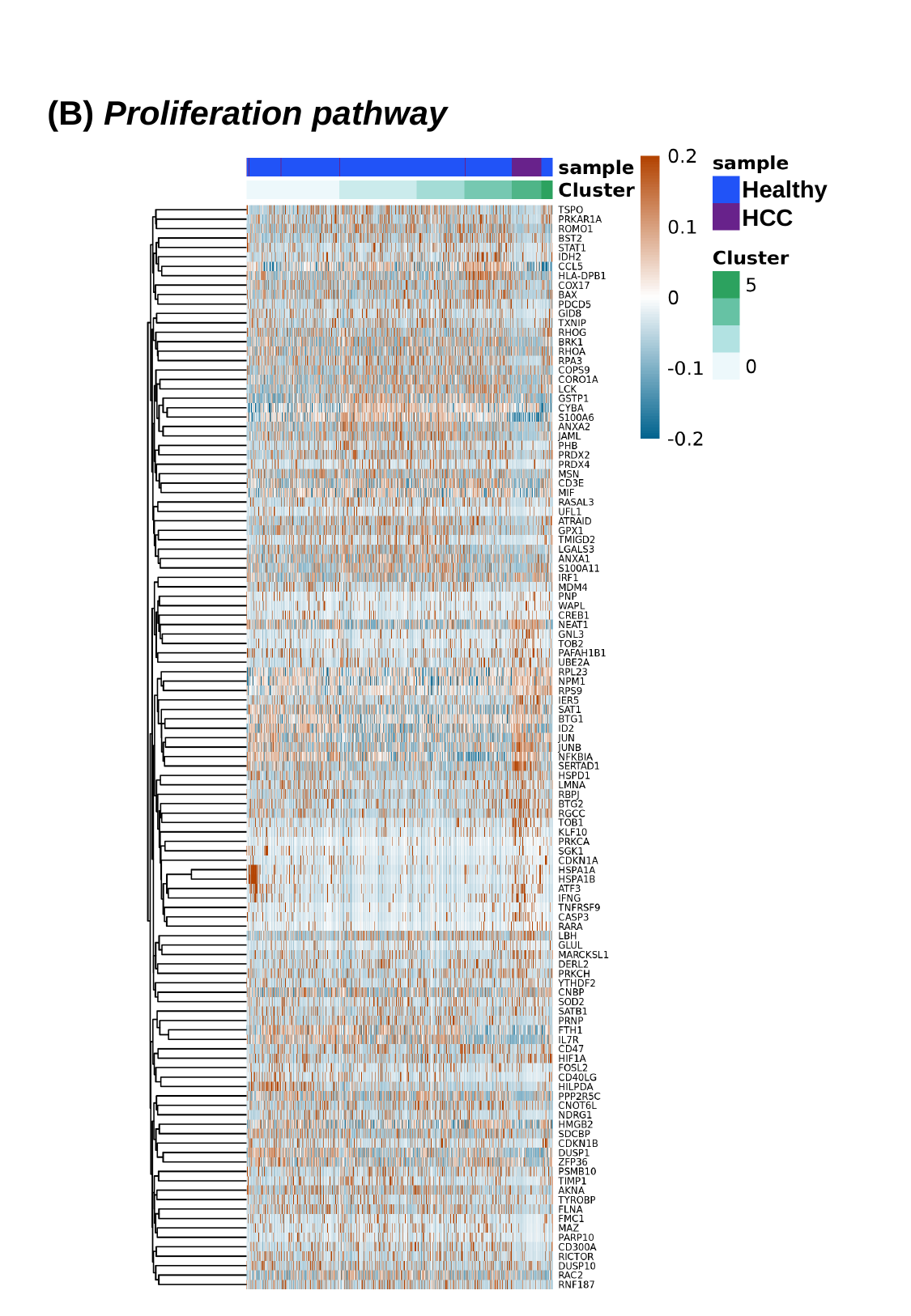

(B) Proliferation pathway
Healthy
HCC

## Slide 9
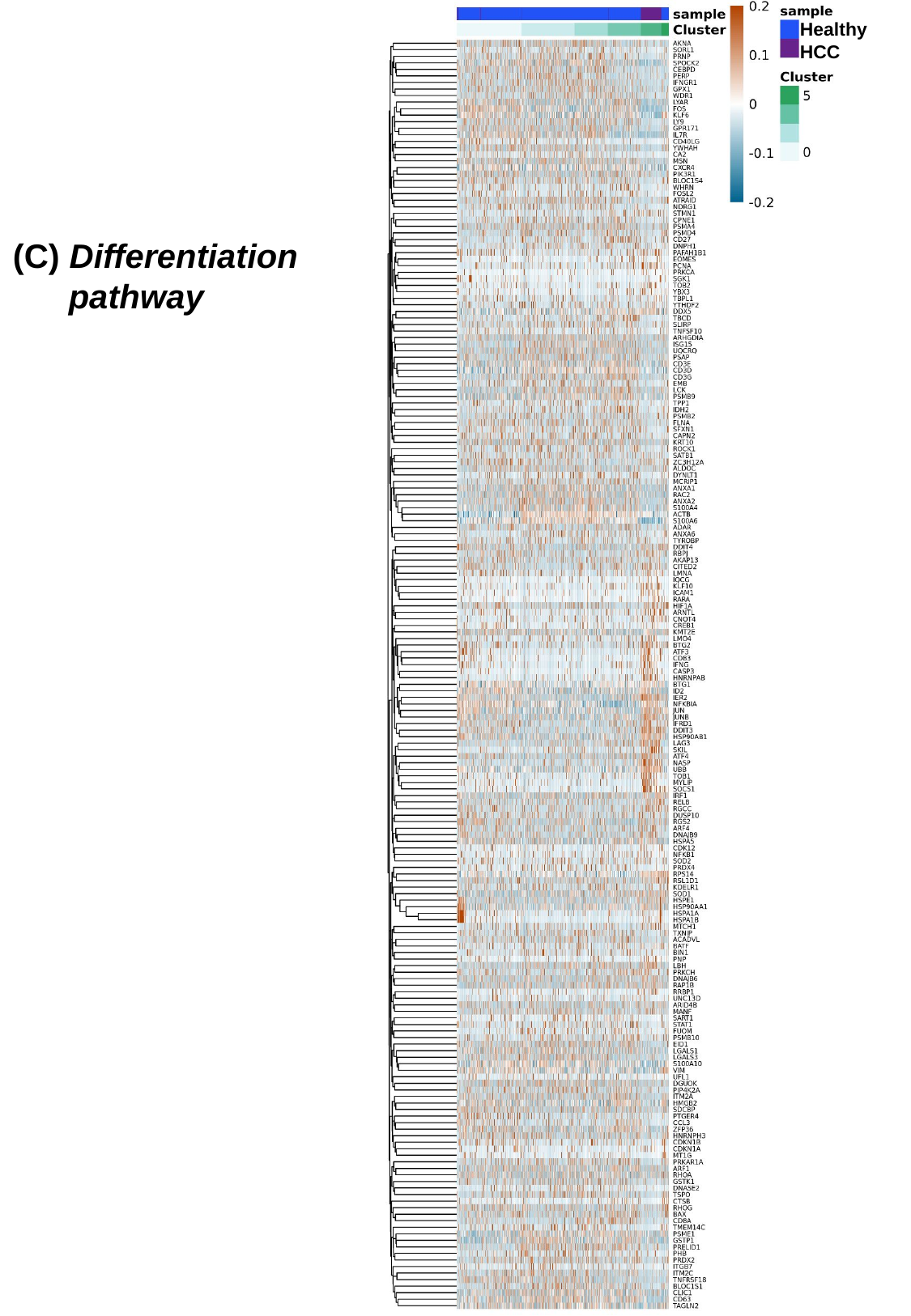

Healthy
HCC
(C) Differentiation
 pathway

## Slide 10
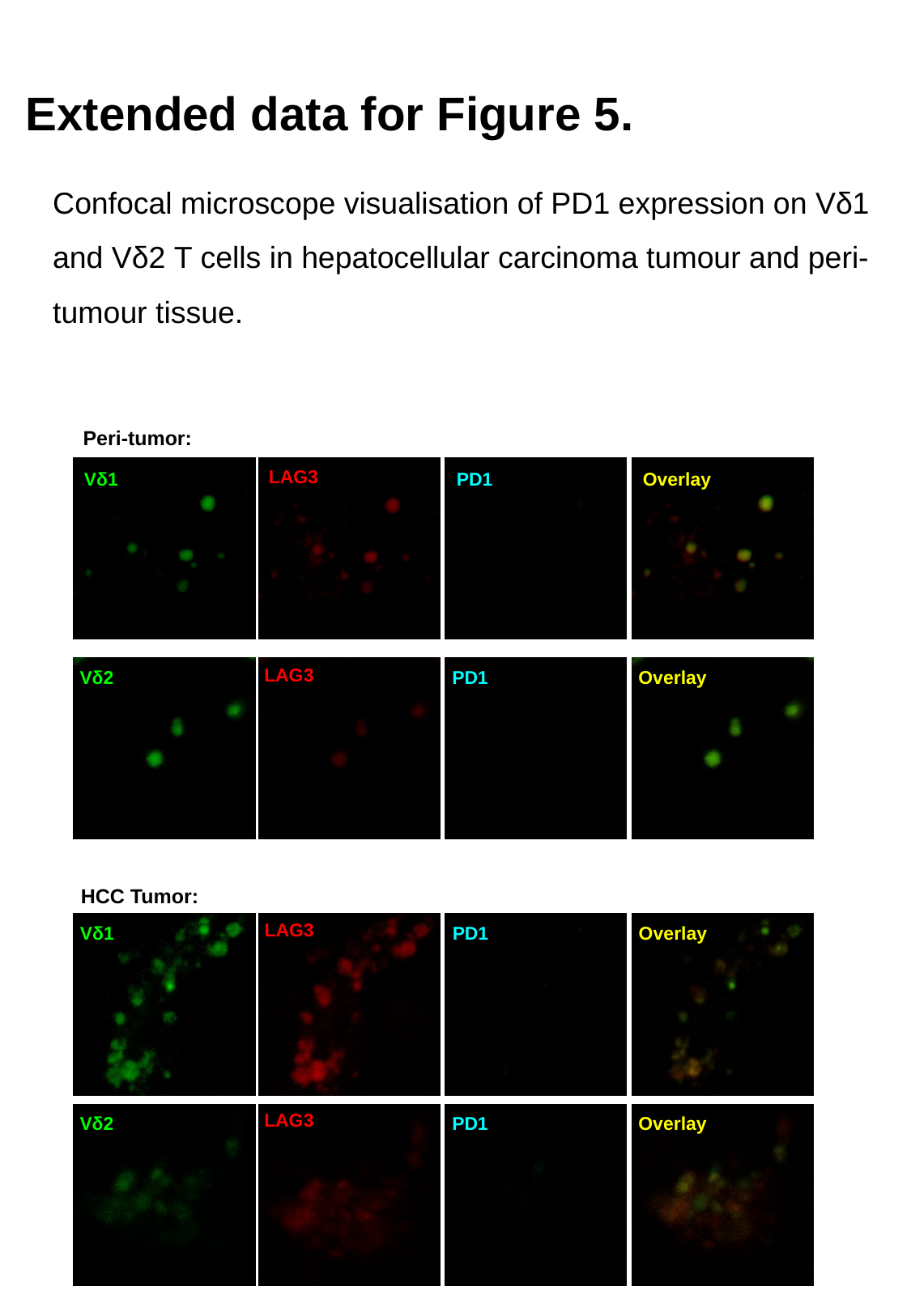

# Extended data for Figure 5.
Confocal microscope visualisation of PD1 expression on Vδ1 and Vδ2 T cells in hepatocellular carcinoma tumour and peri-tumour tissue.
Peri-tumor:
LAG3
Vδ1
PD1
Overlay
LAG3
Vδ2
PD1
Overlay
HCC Tumor:
LAG3
Vδ1
PD1
Overlay
LAG3
Vδ2
PD1
Overlay

## Slide 11
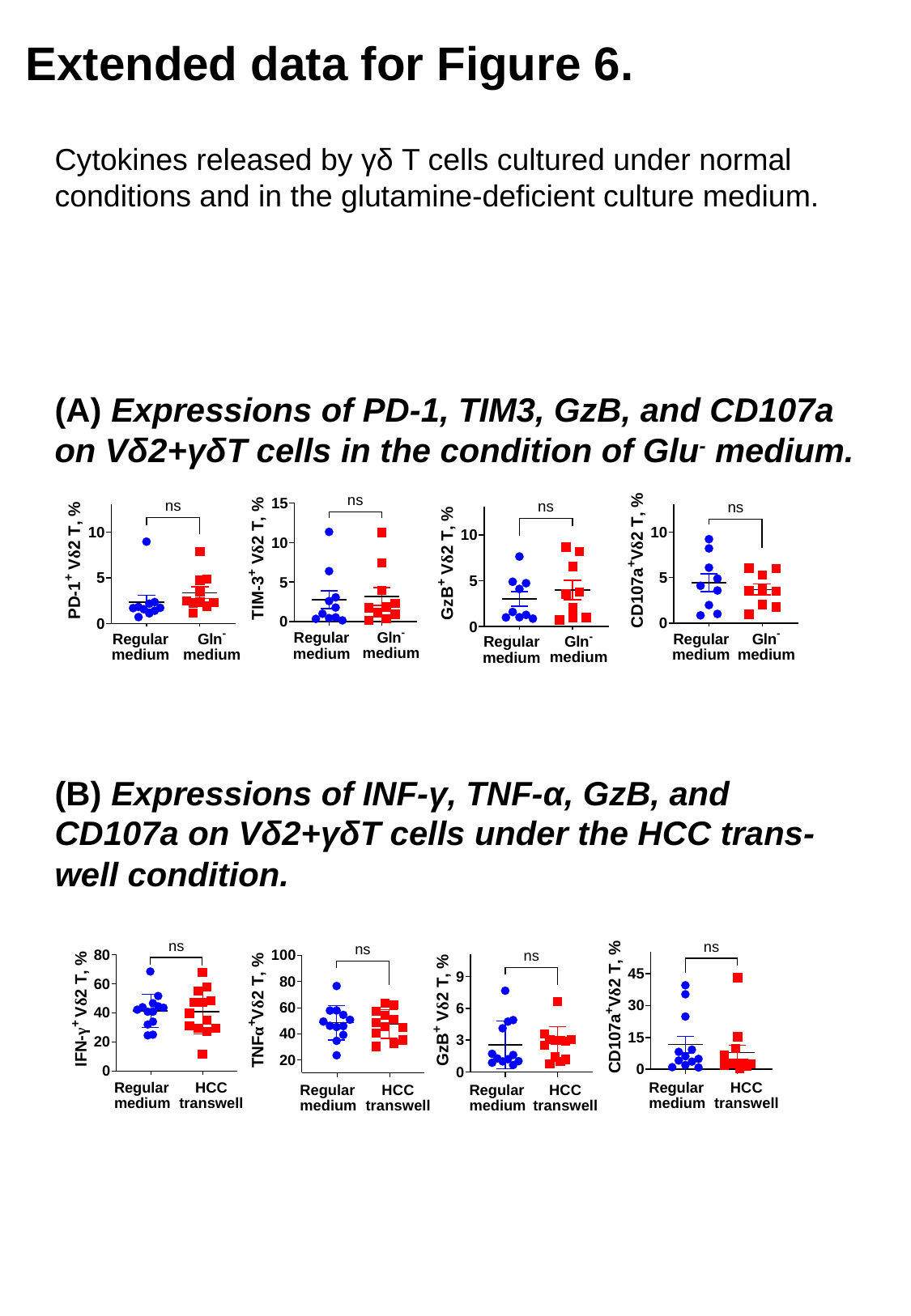

# Extended data for Figure 6.
Cytokines released by γδ T cells cultured under normal conditions and in the glutamine-deficient culture medium.
(A) Expressions of PD-1, TIM3, GzB, and CD107a on Vδ2+γδT cells in the condition of Glu- medium.
(B) Expressions of INF-γ, TNF-α, GzB, and CD107a on Vδ2+γδT cells under the HCC trans-well condition.

## Slide 12
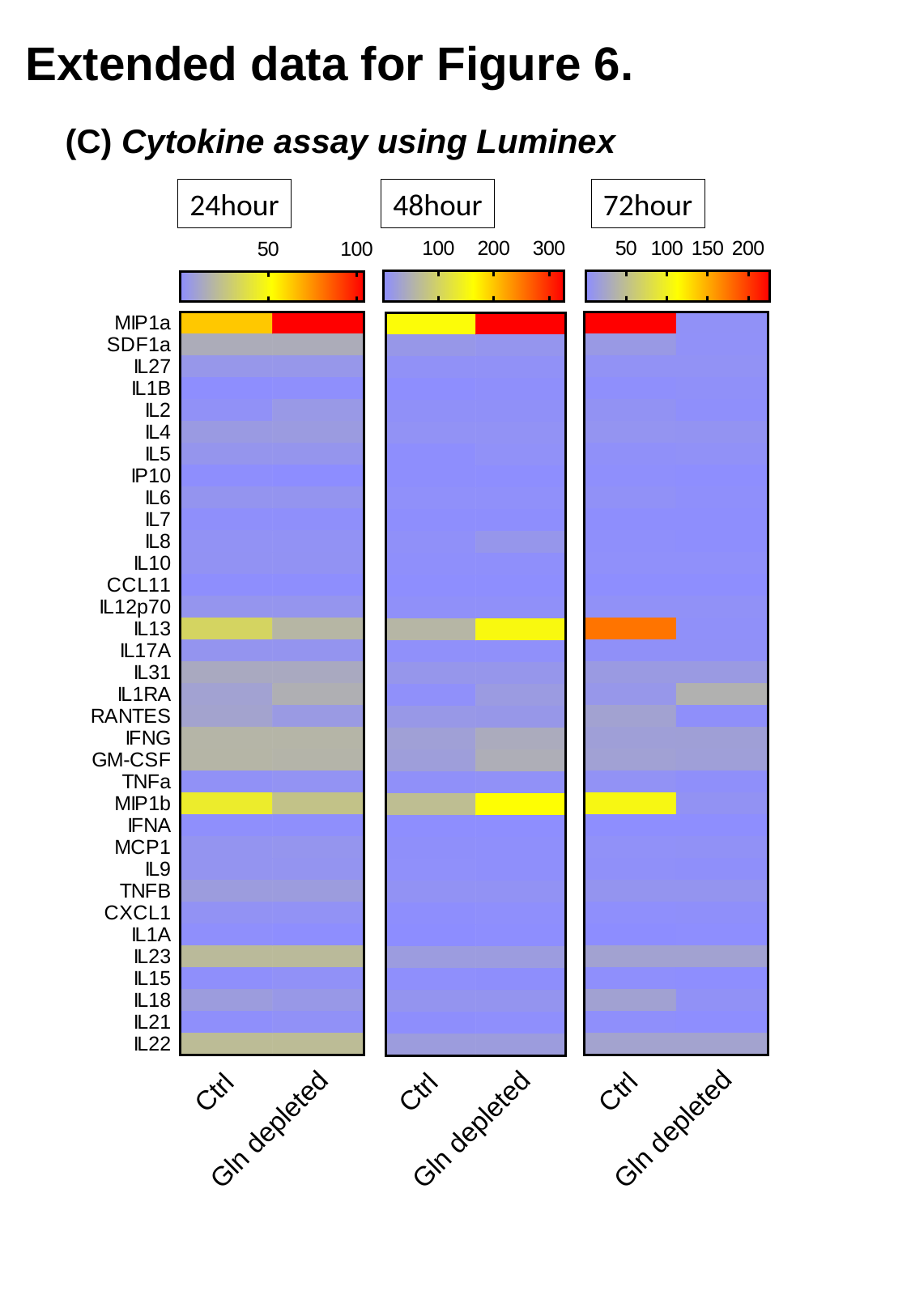

# Extended data for Figure 6.
(C) Cytokine assay using Luminex
24hour
48hour
72hour

## Slide 13
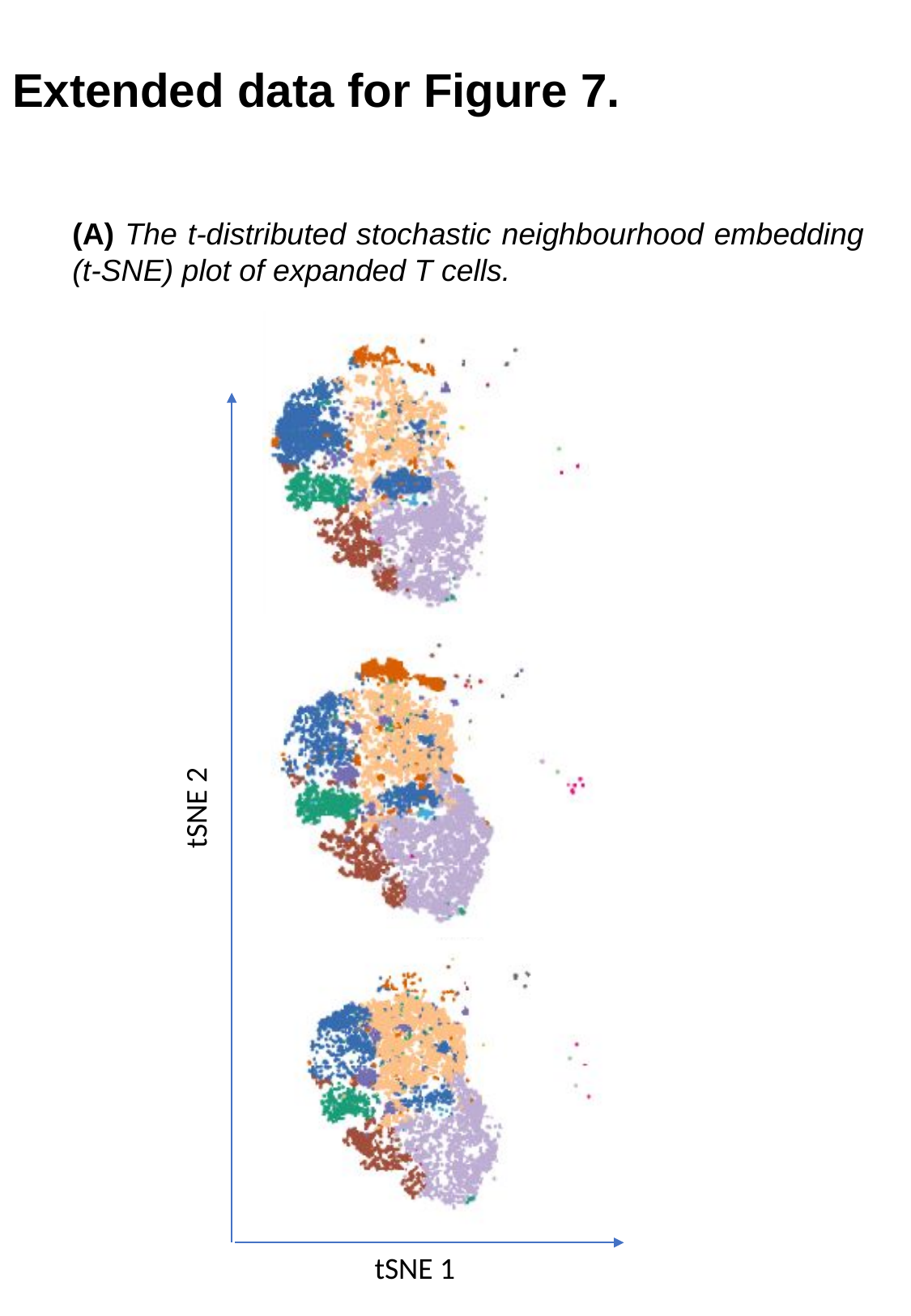

# Extended data for Figure 7.
(A) The t-distributed stochastic neighbourhood embedding (t-SNE) plot of expanded T cells.
tSNE 2
tSNE 1

## Slide 14
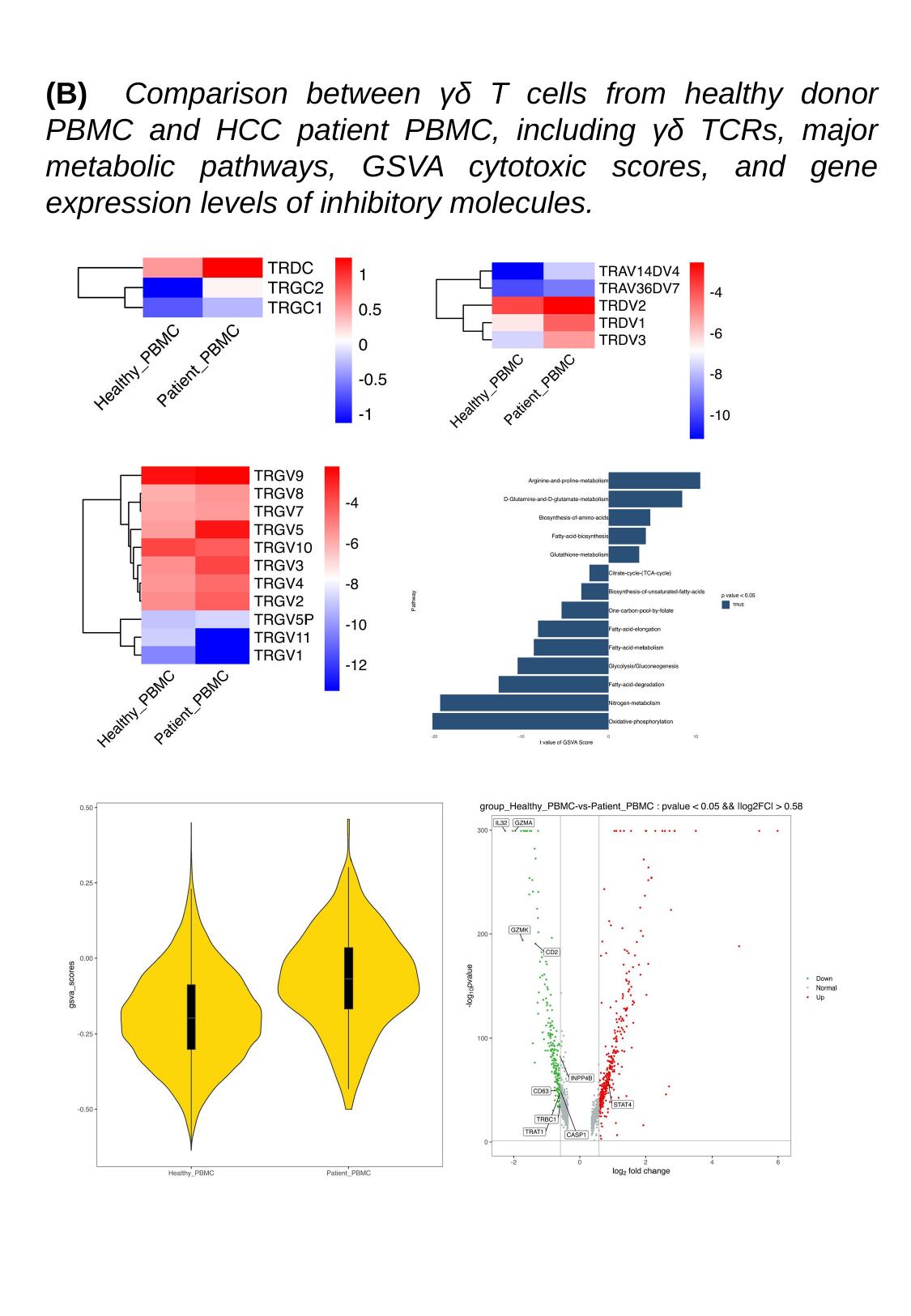

(B) Comparison between γδ T cells from healthy donor PBMC and HCC patient PBMC, including γδ TCRs, major metabolic pathways, GSVA cytotoxic scores, and gene expression levels of inhibitory molecules.

## Slide 15
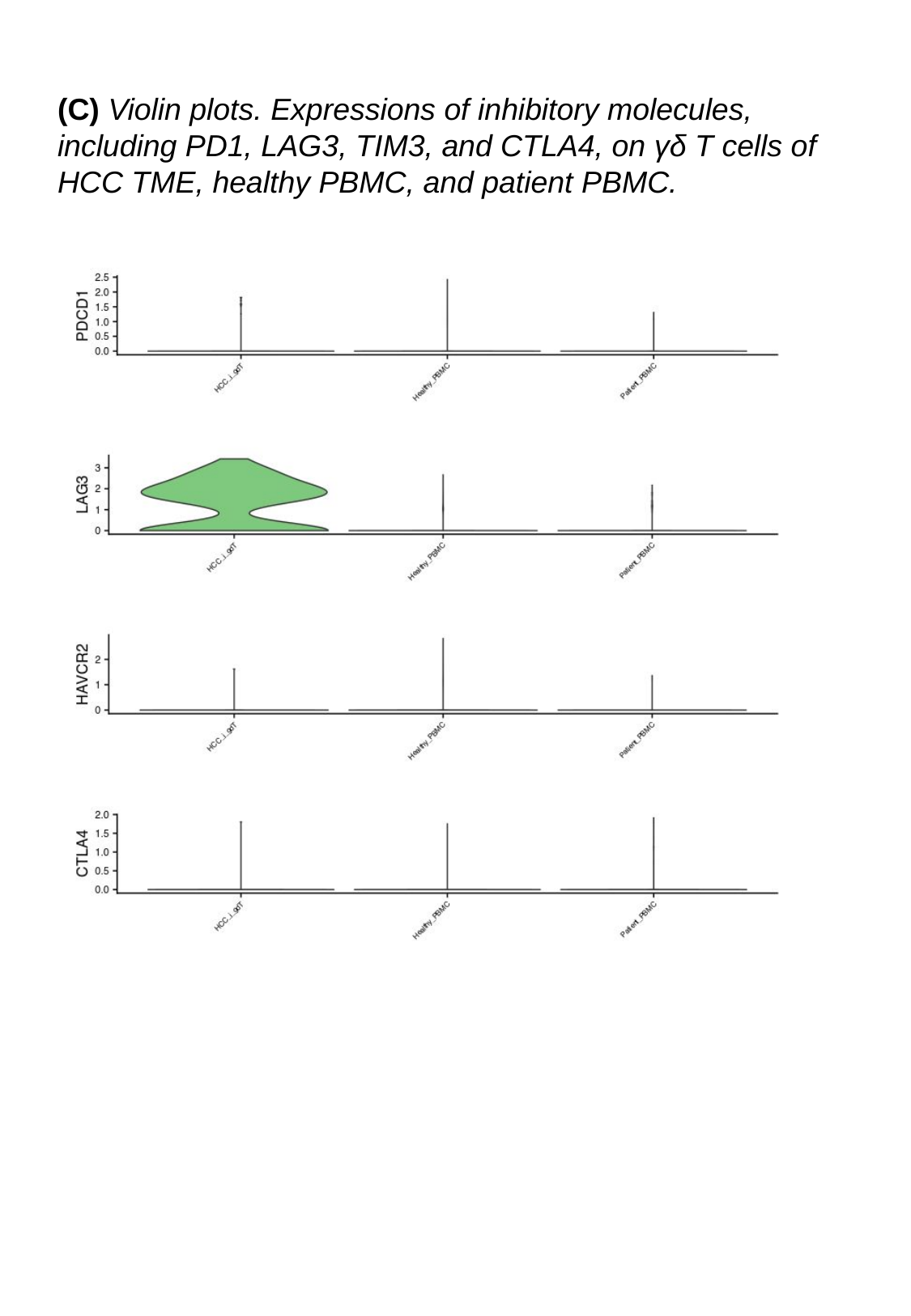

(C) Violin plots. Expressions of inhibitory molecules, including PD1, LAG3, TIM3, and CTLA4, on γδ T cells of HCC TME, healthy PBMC, and patient PBMC.
