# Supplementary material for: Hepatocellular carcinoma‐infiltrating γδ T cells are functionally defected and allogenic Vδ2+ γδ T cell can be a promising complement
Source: Clin Transl Med. 2022 Apr 7;12(4):e800. doi: 10.1002/ctm2.800 (PMC8989380; doi:10.1002/ctm2.800)
Supplement: Supplementary file 4 — Supporting information [file CTM2-12-e800-s004.docx]

**Supplemental Information for**

**‘Materials and Methods’**

***Cell enrichment from liver perfusate and peripheral blood***

To maximally benefit the interests of the patient, it’s difficult to collect sufficient tissue sample from the donated healthy liver during transplantation to match the requirement of 10X sequencing, we thus only collected liver perfusates (before liver transplantation, the liver was maintained in the organ perfusion system) and blood samples from healthy donors. For the purpose of paired comparison, liver perfusates and blood were collected from HCC patients as well. It should be mentioned here that, liver perfusion was considered as one of the main methods to study the heterogeneity of liver resident immune cells [1, 2]. For example, Xystrakis E, *et al.* have demonstrated that intrahepatic lymphocytes obtained through liver perfusion were representative of liver-resident lymphocytes [3, 4]. Importantly, we also tested the reliability of the method perfusion through analyzing γδ T cell proportion. It showed that the obtained γδ T cell proportion in T cells remains no difference between the perfusate samples and the HCC tissue samples (see ‘***Supporting Material (S1)’***). This suggested γδ T cells in liver perfusate share great similarity with the ones in liver tissues. Moreover, we use perfusion since immune cells in perfusate can represent the whole liver resident immune cells, as a contrast, immune cells from tissue sample can only represent local immune microenvironment.

***Single-cell data analysis for scRNA sequencing***

The detailed procedures of sample quality control, sequencing and preliminary analyses and softwares have been extensively described in previous published works ^1-3^. Data analysis mainly includes: **(1)** Cell cycle analysis: cell cycle phase of individual cells was analyzed in Seurat ^1^ with Cell Cycle Scoring function. The gene markers of G2/M and S phases were used to assign cell scores, and cells expressing neither G2/M nor S phase markers were classified as G1 phase. **(2)** Pseudotime analysis: the developmental pseudotime was analyzed using the Monocle2 package ^4^. Gene expression was plotted with the plot_genes_in_pseudotime function to track changes over pseudo-time. **(3)** RNA velocity analysis: the Python script velocyto.py ^5^ (https://github.com/velocyto-team/velocyto.py) and the R package velocyto.R v0.6 ^5^ were used here. **(4)** SCENIC analysis: the database RcisTarget and GRNboost (SCENIC ^6^ version 1.1.2.2, which corresponds to RcisTarget 1.2.1 and AUCell 1.4.1) were used to do analyses. The regulon specificity score (RSS) was analyzed using the Jensen-Shannon divergence (JSD) ^7^. The connection specificity index (CSI) for all regulons was calculated with the scFunctions (<https://github.com/FloWuenne/scFunctions/>) package. **(5)** Cell-cell communication analysis: the CellPhoneDB (v2.0) ^8^ was used to identify biologically relevant ligand-receptor interactions. R packages Igraph and Circlize were used to display the cell-cell communication networks. **(6)** Gene set variation analysis (GSVA): the GSEABase package (version 1.44.0) was used to perform the Gene Set Variation Analysis. To assign pathway activity estimates to individual cells, the GSVA package (version 1.30.0) was applied here ^9^. The differences in pathway activities scored per cell were calculated with LIMMA package (version 3.38.3). **(7)** CNV analysis: the inferCNV ^10^ R package was used for analysis.

***Bulk RNA-seq related procedures***

γδ T cells were firstly ex vivo expanded with regular RPMI1640 medium or glutamine depleted medium. Then total RNA was harvested using RNeasy mini kit (Qiagen, Germany), and the cDNA libraries were created using the TruSeq® Stranded Total RNA Sample Preparation kit (Illumina, USA), according to the manufacturer’s instructions. The libraries were quality controlled by the Qubit® 2.0 Fluorometer (Life Technologies, USA) and then the Agilent 2100 bioanalyzer (Agilent Technologies, USA). Sequencing was conducted using the Illumina HiSeq 2500 (Illumina, USA). The software used in analyses mainly includes Tophat v2.0.9 and Cufflinks v2.1.1.

***Cell sample preparation from tissue samples***

Tissue samples were treated according to the established protocols. In brief, samples were minced on ice to <1 mm^3^ pieces, followed by enzymatic digestion with 1 mg/ml collagenase at 37^o^C for 30 min. Samples were then filtered with Scienceware Flowmi 70-µm cell strainers, and subsequently centrifuged at 300g for 30sec at room temperature to collect the cell pellet, which was then washed with 1×PBS (Ca_2_^+^/Mg_2_^+^ free) containing 0.04% BSA using centrifugation at 300g for 5 min. Red blood cell of the cell pellet was removed using the red blood cell lysis buffer (10min at 4℃).

***γδ T cell functional phenotype by flow cytometry***

Cell surface receptors and intracellular cytokines were stained with flow antibodies following standard staining protocols and then assayed by BD flow cytometry. Flow antibodies mainly include: FITC anti-human CD3 Antibody (BioLegend, HIT3a), APC anti-human CD3 Antibody (BioLegend, HIT3a), V500 Mouse Anti-Human CD3 (BD Biosciences, UCHT1), APC-H7 Mouse Anti-Human CD3 (BD Biosciences, SK7), Brilliant Violet 421™ anti-human CD161 Antibody (BioLegend, HP-3G10), BV510 Mouse Anti-Human Granzyme B (BD Biosciences, GB11), Alexa Fluor® 647 Mouse Anti-Human LAG-3 (CD223)  (BD Biosciences, T47-530), PE anti-human CD223 (LAG-3) Antibody (BioLegend, 11C3C65), PE Mouse Anti-Human Vδ2 TCR (BD Biosciences, B6), PE anti-human TCR Vδ2 Antibody (BioLegend, B6), PE anti-human CD279 (BioLegend, EH12.2H7), Pacific Blue™ anti-human CD279 (PD-1) Antibody (BioLegend, EH12.2H7), BB700 Mouse Anti-Human TIM-3 (CD366) (BD Biosciences, 7D3), FITC anti-human CD366 (Tim-3) Antibody (BioLegend, F38-2E2), PE-Cy™7 Mouse Anti-Human IFN-γ (BD Biosciences, B27), PE anti-human TNF-α Antibody (BioLegend, MAb11), FITC anti-human TNF-α Antibody (BioLegend, MAb11), APC anti-human CD107a (BioLegend, H4A3), PE-Cy™7 Mouse Anti-Human CD107a (BD Biosciences, H4A3), and isotype fluorescent antibodies were used as well.

***Luminex assay***

To examine how glutamine would affect secretions of cytokines and chemokines of γδ T cells, Luminex assay were used here. The Human Cytokine & Chemokine 34-Plex ProcartaPlex Panel 1A kit was used and proceed according to manufacturer’s instructions (Invitrogen, EPX340-12167-901)

***Confocal microscope visualization of infiltrated γδ T cells***

To visually detect infiltrated γδ T cells in HCC tumor tissue and peri-tumor tissue, Leica SP8 confocal microscope system was applied here. Fresh tissue samples were prepared using cryosectioning approach, and then stained with fluorescent antibodies, including anti-human CD3, LAG3, PD1, Vδ1, and Vδ2, by following standard tissue fluorescence staining protocol. Then fluorescence imaging was conducted using the routine procedure.

***Phenotyping peripheral γδ T cells of healthy donors and HCC patients***

To verify the phenotype of peripheral γδ T cells observed in tissue by confocal microscope, we analyzed the data from the database of the Shuangzhi Purui Medical Laboratory Co Ltd (Wuhan, China). The data set contains phenotype information of γδ T cells originated from 54 healthy donors and 20 HCC patients. As for the sample preparation, the Laboratory isolated PBMCs from 2 ml peripheral blood, then stained cells with fluorescence antibody (mouse anti-human γδ TCR, Vδ1 TCR, and Vδ2 TCR) (BioLegend), following by BD FACSCanto flow cytometry analysis. The brief clinical information of these 20 HCC patients is summarized as below:

|  | **HCC Patient Number** | **Gender** | **Age** | **Diagnosis** |
| --- | --- | --- | --- | --- |
|  |  |  |  |  |
| 1 | 508417 | Female | 57 | Hepatocellular carcinoma (IV stage) |
| 2 | 508501 | Female | 71 | Hepatocellular carcinoma (T4N1M0 IV stage) |
| 3 | 507882 | Male | 47 | Low differentiated hepatocellular carcinoma (T4N0M1 IV stage) |
| 4 | 507524 | Female | 56 | Hepatic tubular cell carcinoma (cT3N1M0 IVA stage) |
| 5 | 507606 | Male | 63 | Hepatocellular carcinoma (T3bN1M1 IV stage) |
| 6 | 507590 | Male | 66 | Hepatocellular carcinoma (T3bN1M0 IV stage) |
| 7 | 004701 | Male | 66 | Hepatocellular carcinoma of hepatobiliary duct (T2N0M0 II stage) |
| 8 | 508710 | Male | 60 | Hepatocellular carcinoma (TxN1M1 IV stage) |
| 9 | 507770 | Male | 30 | Hepatocellular carcinoma of hepatobiliary duct (TxN1M1 IV stage) |
| 10 | 503851 | Female | 50 | Hepatocellular carcinoma |
| 11 | 508940 | Male | 47 | Hepatocellular carcinoma of hepatobiliary duct (T4N0M1 IV stage) |
| 12 | 509035 | Male | 56 | Hepatocellular carcinoma |
| 13 | 375046 | Male | 29 | Hepatocellular carcinoma (TxNXM0 IV stage) |
| 14 | 509410 | Female | 40 | Hepatocellular carcinoma (pT4NxMx III stage) |
| 15 | 474048 | Male | 53 | Hepatocellular carcinoma |
| 16 | L28938 | Male | 42 | Hepatocellular carcinoma |
| 17 | 474045 | Female | 62 | Hepatocellular carcinoma |
| 18 | 485827 | Male | 42 | Hepatocellular carcinoma |
| 19 | 509166 | Male | 65 | Hepatocellular carcinoma (T4N1M0 IV stage) |
| 20 | 509331 | Female | 64 | Hepatocellular carcinoma |

References:

1. Butler A, Hoffman P, Smibert P, et al. Integrating single-cell transcriptomic data across different conditions, technologies, and species. Nat Biotechnol 2018;36:411-420.

2. Macosko EZ, Basu A, Satija R, et al. Highly Parallel Genome-wide Expression Profiling of Individual Cells Using Nanoliter Droplets. Cell 2015;161:1202-1214.

3. Aran D, Looney AP, Liu L, et al. Reference-based analysis of lung single-cell sequencing reveals a transitional profibrotic macrophage. Nat Immunol 2019;20:163-172.

4. Trapnell C, Cacchiarelli D, Grimsby J, et al. The dynamics and regulators of cell fate decisions are revealed by pseudotemporal ordering of single cells. Nat Biotechnol 2014;32:381-386.

5. La Manno G, Soldatov R, Zeisel A, et al. RNA velocity of single cells. Nature 2018;560:494-498.

6. Aibar S, Gonzalez-Blas CB, Moerman T, et al. SCENIC: single-cell regulatory network inference and clustering. Nat Methods 2017;14:1083-1086.

7. Suo S, Zhu Q, Saadatpour A, et al. Revealing the Critical Regulators of Cell Identity in the Mouse Cell Atlas. Cell Rep 2018;25:1436-1445 e3.

8. Efremova M, Vento-Tormo M, Teichmann SA, et al. CellPhoneDB: inferring cell-cell communication from combined expression of multi-subunit ligand-receptor complexes. Nat Protoc 2020;15:1484-1506.

9. Hanzelmann S, Castelo R, Guinney J. GSVA: gene set variation analysis for microarray and RNA-seq data. BMC Bioinformatics 2013;14:7.

10. Puram SV, Tirosh I, Parikh AS, et al. Single-Cell Transcriptomic Analysis of Primary and Metastatic Tumor Ecosystems in Head and Neck Cancer. Cell 2017;171:1611-1624 e24.
